# Supplementary material for: Target spike patterns enable efficient and biologically plausible learning for complex temporal tasks
Source: PLoS One. 2021 Feb 16;16(2):e0247014. doi: 10.1371/journal.pone.0247014 (PMC7886200; doi:10.1371/journal.pone.0247014)
Supplement: S1 File — (PDF) [file pone.0247014.s001.pdf]

Target spike patterns enable efficient and biologically plausible learning for complex temporal tasks

Paolo Muratore[1*], Cristiano Capone[2], Pier Stanislao Paolucci[2]

1 SISSA – International School for Advanced Studies, Trieste, Italy
2 INFN, Sezione di Roma, Rome, Italy

These authors contributed equally to this work.
* pmurator@sissa.it

1 Learning Rule for coBa Neurons

The equation for the membrane potential of a conductance based (coBa) neuron can be defined as follows

$$\begin{aligned}\boldsymbol{v}^t = &\left(1 - \frac{\Delta t}{\tau_m}\right)\boldsymbol{v}^{t-1} + \frac{\Delta t}{\tau_m}\left[\boldsymbol{I}^t + v_{rest}\right] \\ &+ \frac{\Delta t}{\tau_m}\left[(E_{\text{exc}} - \boldsymbol{v}^{t-1})\boldsymbol{J}_{\text{exc}}\hat{\boldsymbol{s}}^{t-1}_{\text{exc}} + (E_{\text{inh}} - \boldsymbol{v}^{t-1})\boldsymbol{J}_{\text{inh}}\hat{\boldsymbol{s}}^{t-1}_{\text{inh}}\right] - J_{\text{res}}\boldsymbol{s}^{t-1}\end{aligned} \tag{1}$$

where E_{exc} and E_{inh} are the reversal potentials for excitatory and inhibitory conductances. exc and inh subscripts distinguishes between excitatory and inhibitory neurons and synapses. This means that the populations of neurons has to be segregated in excitatory and inhibitory neurons.

In general it is not straightforward to compare two different models. In order to make a comparison as fair as possible between coBa and cuBa we parametrically defined a coBa model (where $E_{\text{exc}} = v_r$ and $E_{\text{inh}} = -v_r$) that converges to a cuBa model when $v_r \to \infty$. The coBa model can be then rewritten as follows

$$\begin{aligned}\boldsymbol{v}^t = &\left(1 - \frac{\Delta t}{\tau_m}\right)\boldsymbol{v}^{t-1} + \frac{\Delta t}{\tau_m}\left[\boldsymbol{I}^t + v_{rest}\right] \\ &+ \frac{\Delta t}{\tau_m}\left[\frac{(v_r - \boldsymbol{v}^{t-1})}{v_r}\boldsymbol{J}_{\text{exc}}\hat{\boldsymbol{s}}^{t-1}_{\text{exc}} + \frac{(-v_r - \boldsymbol{v}^{t-1})}{v_r}\boldsymbol{J}_{\text{inh}}\hat{\boldsymbol{s}}^{t-1}_{\text{inh}}\right] - J_{\text{res}}\boldsymbol{s}^{t-1}\end{aligned} \tag{2}$$

We derive the spike response functions for excitatory and inhibitory weights:

$$\nabla_{\boldsymbol{J}_{\text{exc}}}\boldsymbol{v}^t = \left(1 - \frac{\Delta t}{\tau_m} - \frac{\Delta t}{\tau_m}\frac{|\boldsymbol{J}|}{v_r}\hat{\boldsymbol{s}}^{t-1}\right)\nabla_{\boldsymbol{J}_{\text{exc}}}\boldsymbol{v}^{t-1} + \frac{\Delta t}{\tau_m}\frac{(v_r - \boldsymbol{v}^{t-1})}{v_r}\hat{\boldsymbol{s}}^{t-1}_{\text{exc}} \tag{3}$$

and

$$\nabla_{\boldsymbol{J}_{\text{inh}}}\boldsymbol{v}^t = \left(1 - \frac{\Delta t}{\tau_m} - \frac{\Delta t}{\tau_m}\frac{|\boldsymbol{J}|}{v_r}\hat{\boldsymbol{s}}^{t-1}\right)\nabla_{\boldsymbol{J}_{\text{inh}}}\boldsymbol{v}^{t-1} + \frac{\Delta t}{\tau_m}\frac{(-v_r - \boldsymbol{v}^{t-1})}{v_r}\hat{\boldsymbol{s}}^{t-1}_{\text{inh}} \tag{4}$$

Since in the coBa it is necessary to separate excitatory and inhibitory neurons, in our comparison, we did the same for the cuBa network.

It follows the result of our comparison between coBa ($v_r = 10$) and cuBa ($v_r \to \infty$) on the task of learning a 3D trajectory (the parameters are the same as in Fig1 except, $time = 0.1s$, $\sigma_{train} = 1$). The thin lines represent the spike error $\Delta S = \frac{1}{NT}\sum_{it}|s^t_{i,\text{targ}} - s^t_{i,\text{pred}}|$ for the single experiment, while the thick line is the average over 10 experiments. coBa and cuBa performances are reported in black and red respectively. The performances of the two networks are comparable in terms of error convergence.

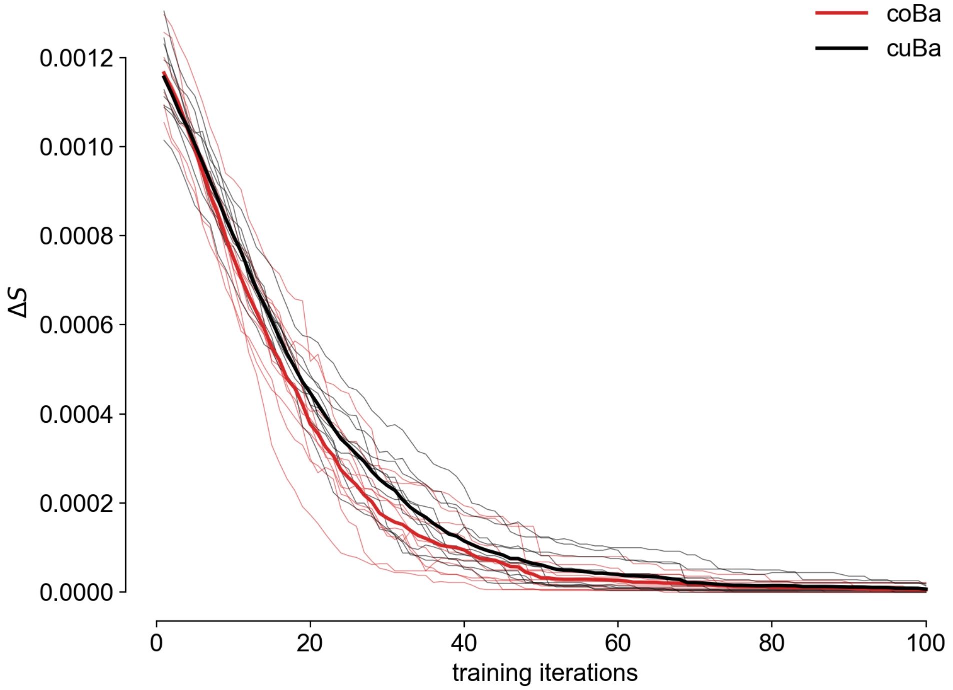

Fig S1. Comparison of cuBa and coBa. Direct comparison of the dynamics of training of the cuBa and coBa neuron model. The number of erroneous spikes with respect to given target is reported as a function of the iteration of training. Thin red and black are single trial of cuBa and coBa models respectively. Solid thick lines are training averages.

2 Learning with different τ_m

In this section we investigate the dependence of a different membrane timescale of the learning speed. The membrane time constant τ_m intuitively modulates the inertia to changing membrane potential due to incoming stimuli: low timescales correspond to fast-responding neurons. To investigate the effect of this quantity on the learning dynamics, we compare several instances of our model trained on the same task (3D trajectory generation) with varying τ_m. Results of this investigation are reported in Figure S2.

Experimentally we showed that decreasing the time constant improves convergence speed.

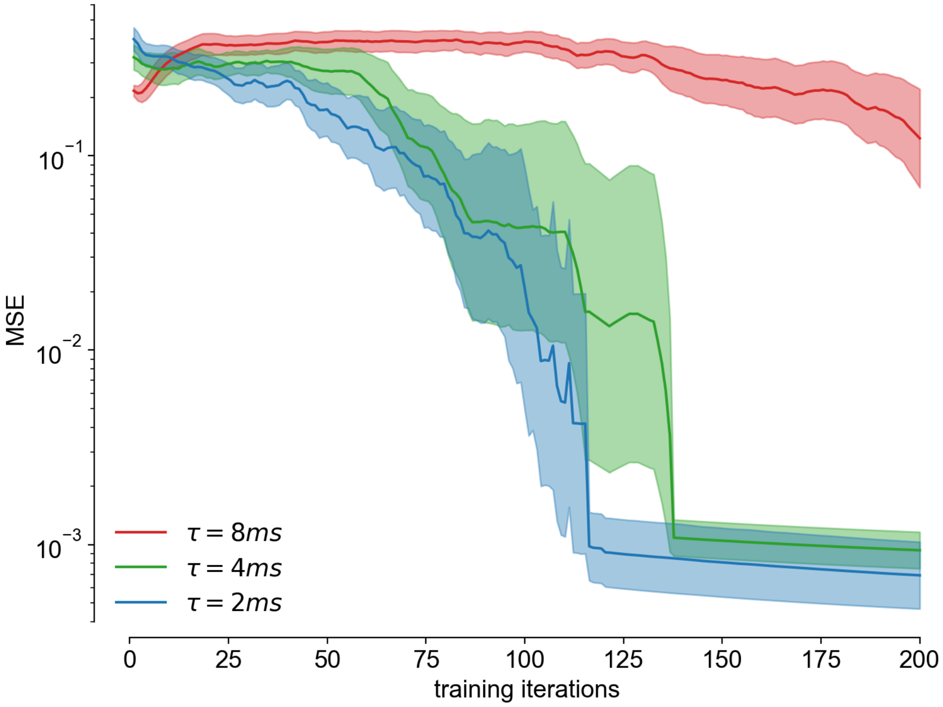

Fig S2. Effect of τ_m of learning speed. A direct comparison of the training dynamics for the 3D trajectory task for different membrane timescales. Longer timescales corresponds to longer training convergence.

3 Gradient ascent vs online approximation

Here we validate that the beneficial effects of training with online approximation as opposed to gradient ascent are indeed conserved and even enhanced for more complex tasks. We show that the effect is not a consequence of smaller timescales.

In Figure S3 we prove that online approximation is beneficial to learning convergence speed even for complex temporal task. We test out system for the 3D trajectory generation task for network dimensions $N = 500$, $T = 1000$ and again models trained with online approximations showed significant improvement as opposed to and exact (gradient ascent) evaluation of the likelihood gradient.

This evidence (together to the results described in the Main Text) strengthen the conclusion that indeed computing an online approximation of the likelihood gradient is not only required for biological plausibility, but is beneficial in terms of learning speed. We leave the theoretical exploration of this effect for future works.

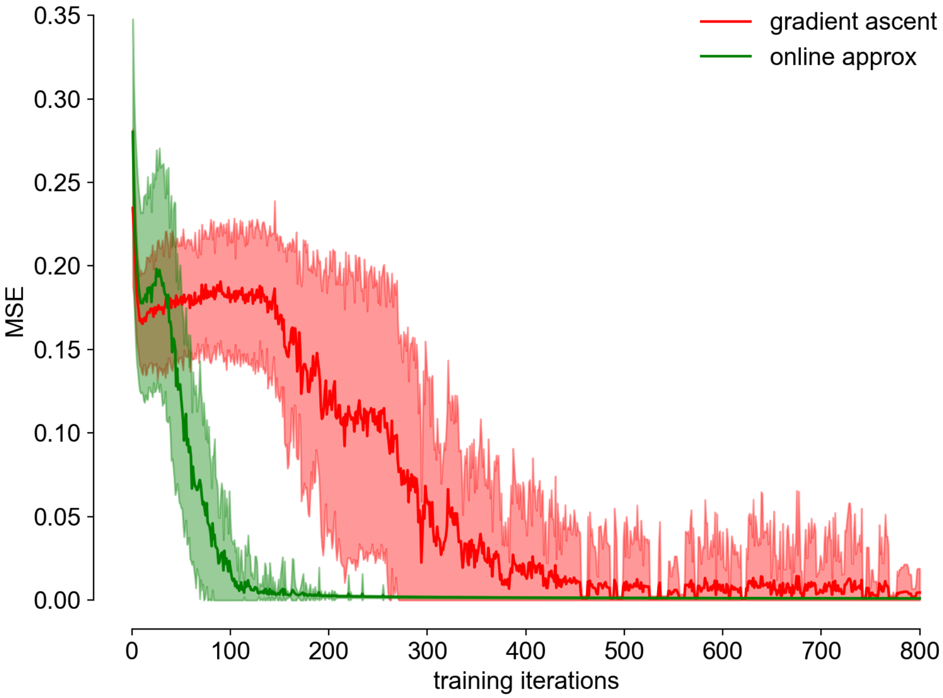

Fig S3. Validation of Online Training for Complex Tasks. Direct comparison of training dynamics on 3D trajectory task for the gradient ascent (red line, shaded area represents one standard deviation over 100 realization) and its online approximation (green line, shaded area represents one standard deviation over 100 realization).

4 Learning without Clock

In this section we provide evidence of the fact that our model doesn't need an external clock to successfully complete the standard 3D trajectory task. Indeed in the Main Text the external clock was primarily introduced to exactly reproduce the given task thus enabling exact comparisons with other algorithms.

However in this case the processing of an input is not required, and the trajectory can be store and recalled without a clock current. The recall of the sequence can be triggered by inducing in the network the first spikes of the spike pattern coding for the 3D trajectory (initialization phase, see S4).

In S4(bottom) we reported an example of the internal activity of 50 randomly extracted neurons. The correct pattern of spikes is spontaneously generated by the network after the initialization phase.

In S4(top) we reported the readout of one of the trajectories together with the achieved MSE. Our model achieves very accurate performances even in the absence of a guiding clock input.

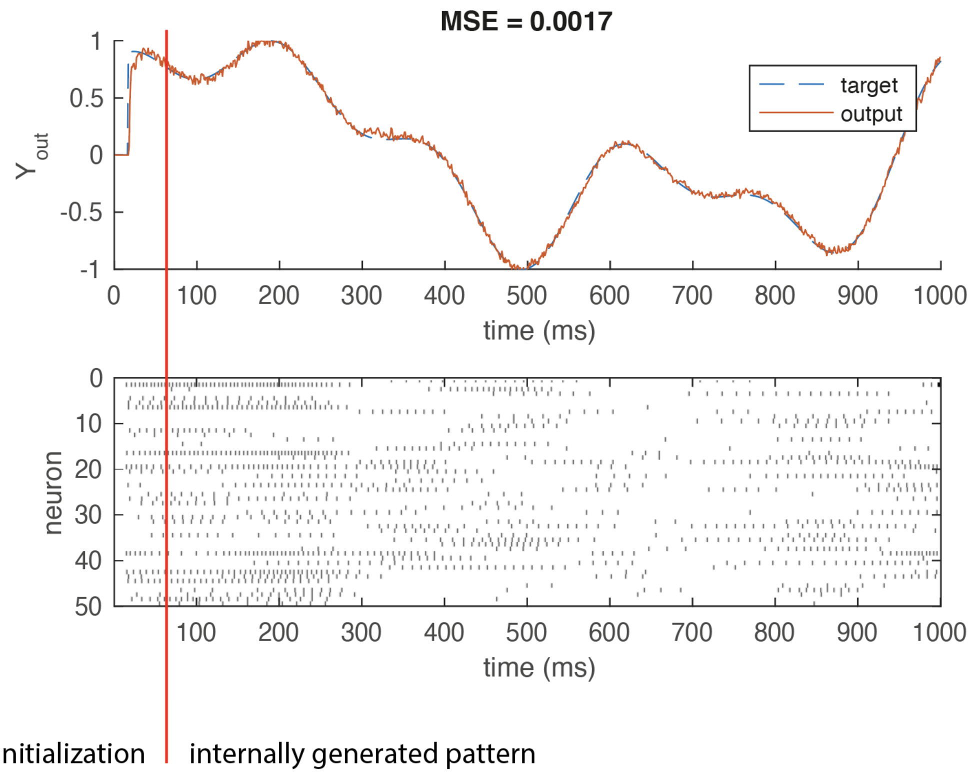

Fig S4. Learning without clock. (top) Example of one of the retrieved output trajectory: blue is the target, orange is the extracted readout. (bottom) Representation of the internal network dynamics for a random sub-population of 50 neurons in the absence of an external clock signal.

5 Comparison of training dynamics for different δv

In this section we offer a direct comparison between the performances of our model for different choices of the δv parameter in the standard 3D trajectory task.

In Figure S5 we compare the training dynamics measured by the MSE computed on the output trajectories. All three choices for the parameters achieved the same final MSE but differences emerged on the early training phase. We achieved maximum convergence speed for $\delta v = 0.2$.

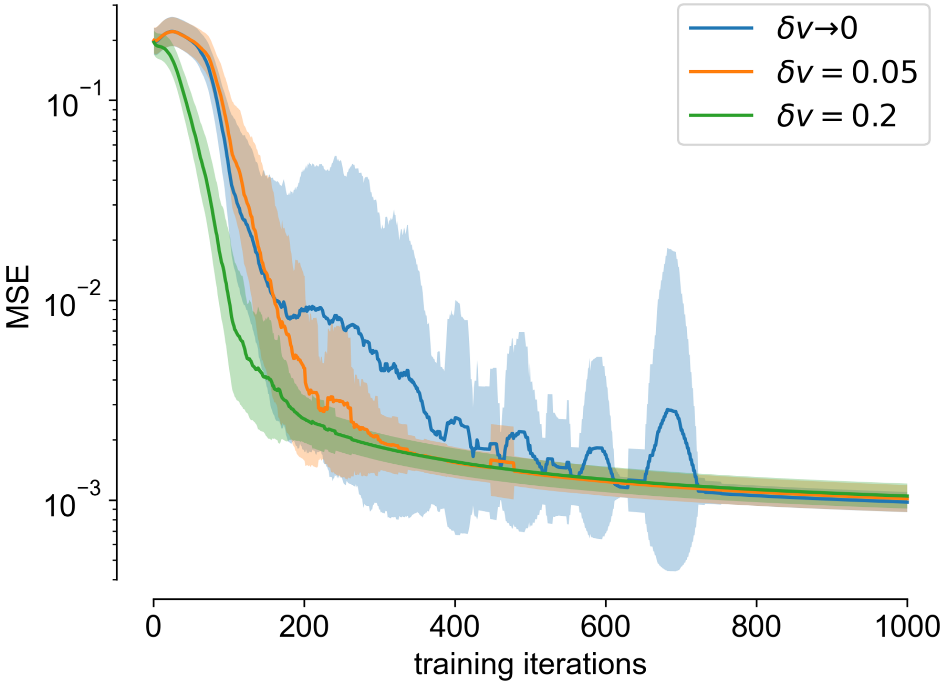

Fig S5. Comparison of training dynamics for different δv. Training trajectories comparison between three possible choices for the δv parameter on the standard 3D trajectory task (see Main Text for further description of the task). Parameters of the models (other than δv) as described in the Main Text.

6 Stability under perturbation of initial conditions

In this section we explore the stability of our model under perturbation of the initial condition of the membrane initial potential $v_0 = v_{\text{rest}}$. A plausible model for realistic biological computation should be robust under noise perturbation: in the main text we offered evidence of robustness under input current corruption, here we demonstrate that indeed optimal performances do not rely critically on a precise initial condition (for example $v_0 = v_{\text{rest}}$ for each unit). We test the behaviour of our model on the 3D trajectory task when initialized, during testing, with a random initial condition of the form $v_0 = v_{\text{rest}} + \xi$, where $\xi \sim \mathcal{N}(0, \sigma_{\text{init}})$.

In Figure S6 we report both the MSE (computed on the output 3D trajectory) and the $\Delta S = \frac{1}{NT} \sum_{it} |s^{t+1}_{i,\text{targ}} - s^{t+1}_{i,\text{pred}}|$ as a function of $\sigma_{\text{init}} / (v_{\text{th}} - v_{\text{rest}})$. Indeed we find that our model do posses a region of stability, after which the increase in output MSE is smooth.

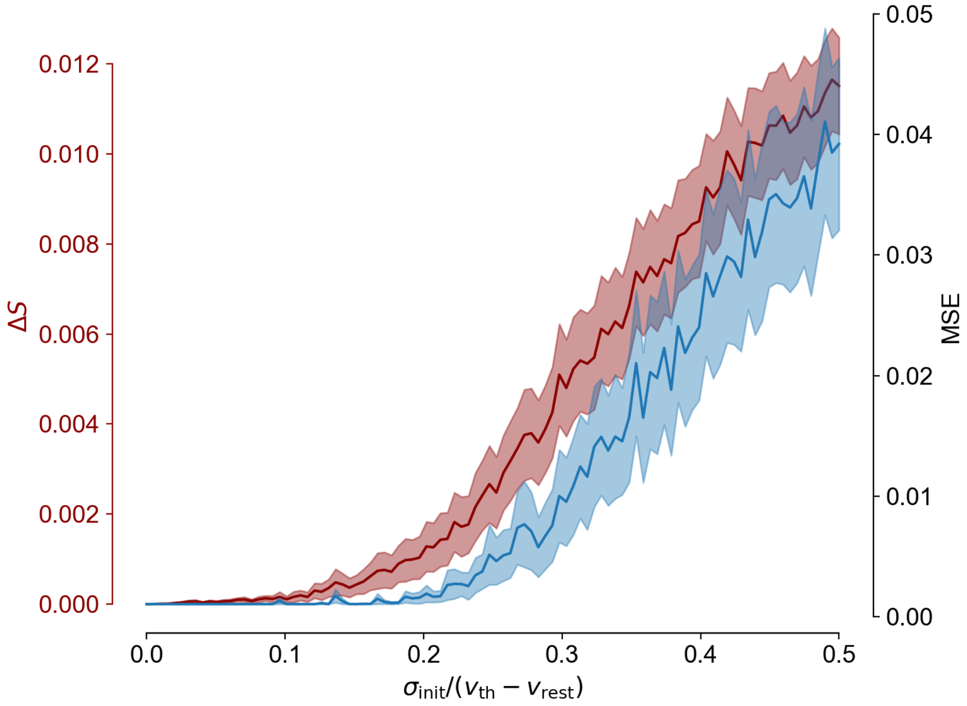

Fig S6. Robustness to noise on initial conditions Output MSE and ΔS as a function of the amount of noise on the initial condition measured as $\sigma_{\text{init}} / (v_{\text{th}} - v_{\text{rest}})$. Each point is obtained as the average over 25 realization of the random noise, solid lines are averages over 25 trained models. Shaded area is std$/\sqrt{N_{\text{train}}}$, where $\text{N}_{\text{train}} = 25$. Model parameter are the one described in the Main Text for the 3D trajectory task for $\delta v = 0.05$.

7 Network Capacity: Parity Check Task

In a recurrent network, subject to time-structured input current and facing an arbitrary task, it is not easy to define an objective notion of network capacity. Nevertheless we propose here an heuristic answer to such question.

We considered the following parity check task, which is the natural extension of the XOR for more than two inputs. The network receives a stream of n_b bit (encoded as either long or short square pulses) and is asked to produced the parity bit (1 if the number of received 1 is odd, 0 otherwise) at the end. The network answer is coded as either a positive (parity bit is 0) or negative (parity bit is 1) response, similarly to the **Temporal XOR** section of the main text. When $n_b = 3$ the possible input combinations are 8, or 2^{n_b} for the generic case. We can use this task to probe the network response accuracies beyond the $n_b = 2$ case of the temporal XOR. The network capacity can thus be heuristically associated to the *failing point* or maximal number of correct answers $n_{\max}$ obtained for the parity check task, when the network size N is fixed and n_b is progressively increased.

When we tested our system we obtained that the maximum number of correct answer was $n_{\max} = 6$ for $N = 500$ (trained for 1000 epochs with Adam optimizer, where each epoch corresponds to the presentation of the randomly-shuffled complete set of patterns). We tried to go further and included a new feature in the model. Indeed, in this case, when the temporal computation required to the network increases, it is difficult to achieve very good results in absence of recurrent connections when the target is generated. For this reason we introduced recurrent weights between the apical compartments of the neurons. This only changes the way the target pattern of spikes s_{targ} is evaluated, which is indeed done under the influence of these recurrent apical connections (while for all the other experiments, s_{targ} was computed by removing all the recurrent weights). We remark that these recurrent apical connections are not plastic and are not influenced by the training. Upon initialization, they are drawn from a Gaussian distribution $J_{ij}^{\text{ap}} \sim \mathcal{N}(0, \sigma_{\text{ap}})$, with $\sigma_{\text{ap}} = 1.5$, and remain fixed throughout the subsequent computations. Using this new definition of the target pattern of spikes s_{targ} we obtain better results and all the 8 patterns of the $n_b = 3$ parity are correctly classified (after 1000 epochs of training with Adam optimizer, where each epoch is defined as the presentation of completed - randomly shuffled - set of patterns). We also tried the $n_b = 4$ parity: 14 out of the 16 patterns are correctly classified (trained for 1000 epochs with Adam optimizer).

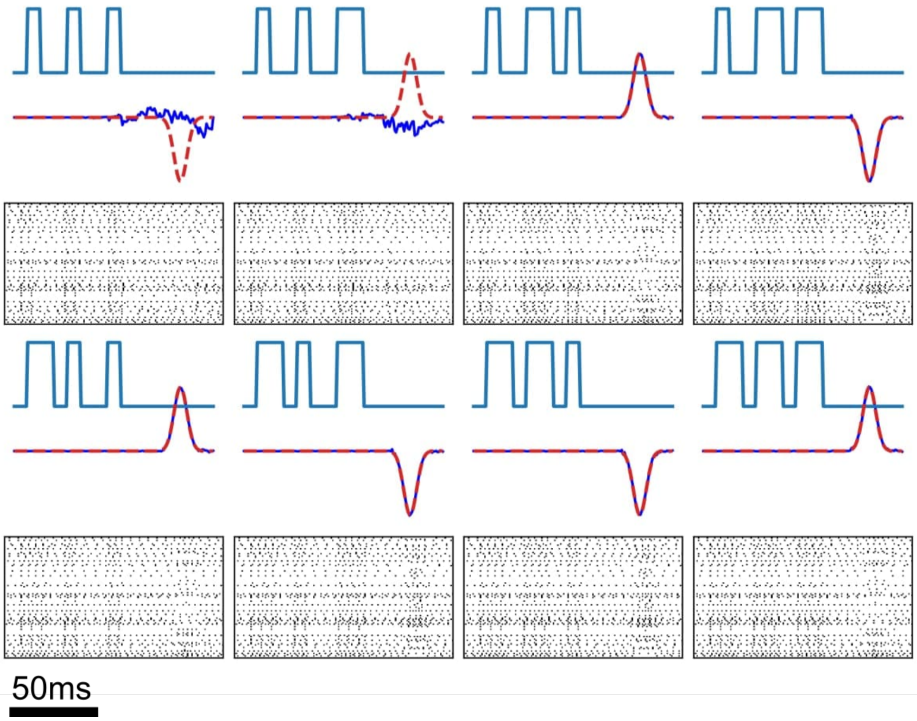

Fig S7. Parity Check Results for the parity check task for $n_b = 3$ when no recurrent apical connections are used in the s_{targ} computation. The network classified 6 out of 8 cases correctly for $N = 500$.

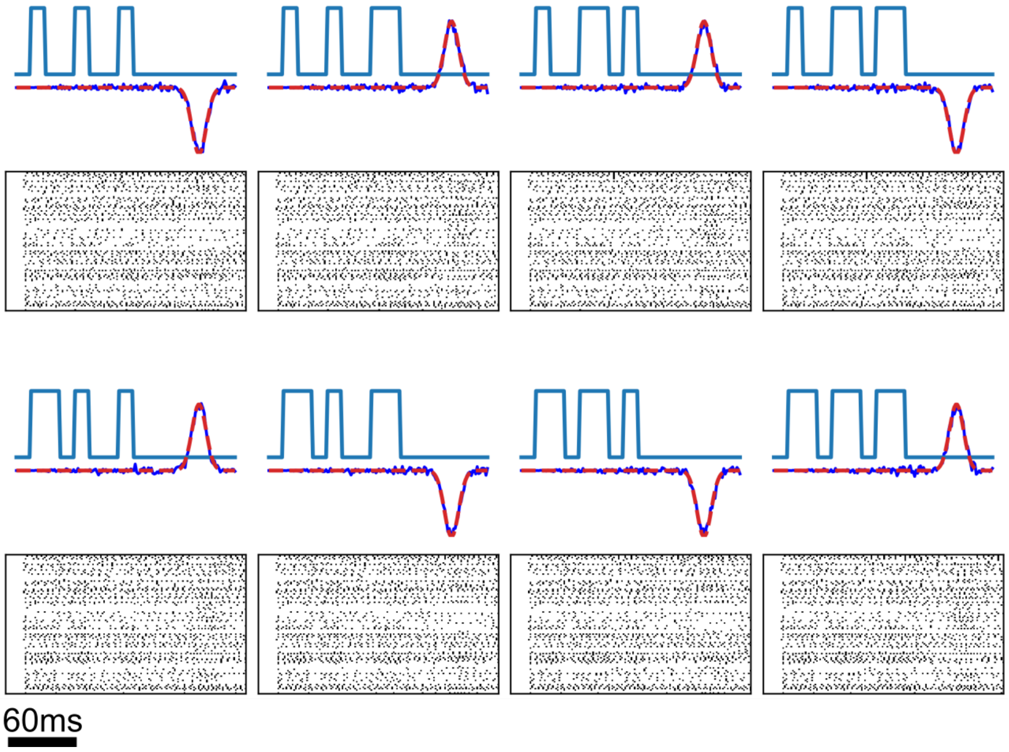

Fig S8. Parity Check with Recurrent Apical Connection $n_b = 3$. Results for the parity check task for the $n_b = 3$ case when the network target patterns s_{targ} are produced using recurrent apical connections. The network solves the case $n_b = 3$ perfectly with $N = 500$.

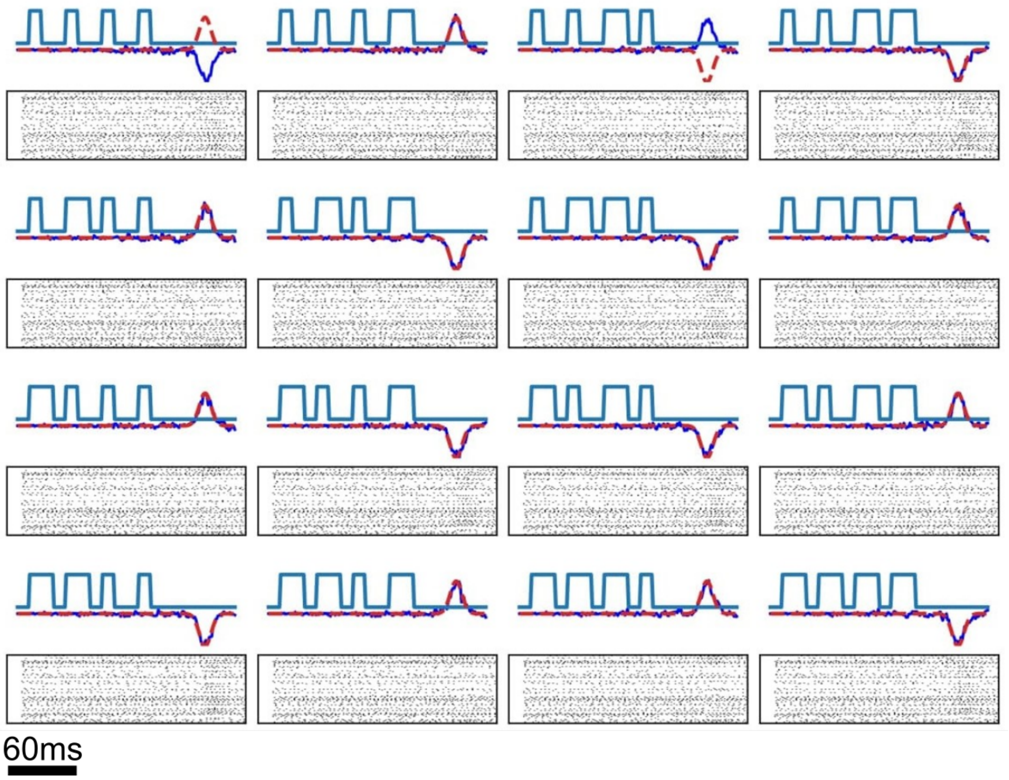

Fig S9. Parity Check with Recurrent Apical Connections $n_b = 4$. Results for the parity check task for the $n_b = 4$ case when the network target patterns $s_{\rm targ}$ are produced using recurrent apical connections. The network correctly responded in 14 out of 16 cases and network size $N = 500$.
