# Supplementary material for: Target spike patterns enable efficient and biologically plausible learning for complex temporal tasks
Source: PLoS One. 2021 Feb 16;16(2):e0247014. doi: 10.1371/journal.pone.0247014 (PMC7886200; doi:10.1371/journal.pone.0247014)
Supplement: Attachment — Submitted filename: Reply to reviewers - PONE2020.pdf [file pone.0247014.s003.pdf]

Reply to Reviewers

Target spike patterns enable efficient and biologically plausible learning for complex temporal tasks

Paolo Muratore, Cristiano Capone, Pier Stanislao Paolucci

Editor 1

Reviewer #1 6

Reviewer #2 18

Reviewer #3 26

Editor

Thank you for submitting your manuscript to PLOS ONE. After careful consideration, we feel that it has merit but does not fully meet PLOS ONE's publication criteria as it currently stands. Therefore, we invite you to submit a revised version of the manuscript that addresses the points raised during the review process.

The manuscript should be revised for clarity and address concerns of the reviewers about details of the algorithm. Suggested literature should be discussed. The following specific concerns should be addressed.

The Authors claim to use leaky integrate and fire neurons (see Eq. (3)), but in the discussion no reset of v_t is mentioned. Such resets, while being a potentially essential source of non-linearity in the dynamics of the network, could be problematic when calculating derivatives of v_t with respect to J. Please, discuss this issue.

After the spike, a negative term (- J_res * s_ii^t) affects the neuron provoking the after spike reset of the membrane potential. We mistakenly did not include it in the text. We corrected the equation including such a term (see new eq(4) and (13)).

Such formalization does not have derivability issues. The derivative of v_t with respect to the weights is defined iteratively (see new eq(18)). The reset induced by the spikes doesn't depend on J and doesn't appear in the expression of V_t/ dJ.

The Authors claim that their method is biologically plausible. However, equation (16) is recursive and requires the storage of the full history of membrane potentials and spikes throughout the training. This does not seem to be biologically plausible (two phases needed, and how are the values stored in the biological substrate?) and may be impractical in engineering applications. It is not clear whether the online version of the algorithm given by (6) alleviates these problems,

because not enough details of this procedure are currently given. For example, are targets still generated during a separate phase in the online training paradigm?

Eq(16) (new eq(18)) only depends on the previous time step and no storage is required.

Indeed, in the simple gradient version of the learning, at every time dV_t/dJ can be evaluated from the previous time step, and a partial update DJ_t = (S_targ-S_pre) dV_t/dJ is evaluated. The partial updates for each time are accumulated until the end of the trial, after which the weights are eventually updated. This only requires the accumulation of partial updates.

The online approximation consists in performing online each partial update. This makes the accumulation not necessary.

Another point is the storing of the target pattern s_targ. Indeed, we evaluate it in a first phase and store it. This is true both for the simple gradient and the online approximation.

However, it is possible (and absolutely equivalent) to evaluate online this pattern at every trial. Indeed, the target pattern is the one generated by the teaching signal in absence of recurrent synaptic connection. This might be evaluated online by a dedicated compartment of the neuron that receives connections from the teacher and doesn't receive recurrent connections.

We claim this to be biologically plausible: in pyramidal neurons, it is known that the apical and basal compartment receive respectively contextual (or teaching) and sensory inputs [Larkum2013].

We explain better this point in the text:

"In terms of biological plausibility, we can assume that the target pattern of spikes is computed by a dedicated compartment of the neuron that receives the training current.

This would be totally equivalent to our protocol. It can be locally computed at every trial of the training procedure, making always accessible the target sequence to the network, without the requirement of an expensive storing of the target. We consider this assumption biologically plausible because of the increasing consensus on the specific role of the apical compartments of pyramidal neurons [Larkum2013] which receives contextual signals from other areas."

-Please, clarify how the 'internal target spike trains' are obtained. How are the spike trains obtained from a teaching input current I_teach?

The target pattern of spikes is the pattern induced by I_teach in the network in absence of recurrent synapses. In the end, the learning of the recurrent synapsis should allow the network to recall the spiking target in absence of I_teach.

We clarified such point in the manuscript (line 107):

"We define the internal target $s_{i, \mathrm{targ}}^t$ as the pattern of spikes induced in the network by the training current $\bm{I}_\mathrm{teach}^t$, in absence of recurrent connections, possibly in combination with an additional input signal that, depending on the task, can be either

superfluous or essential. In the memory recall tasks the latter may serve as an external clock, in others (e.g. XOR task) it is the input to be processed to solve the task."

-The teaching current is projected randomly to all the neurons in the network. What if some neurons do not receive a teaching current? In Brea et al. there is a pool of hidden neurons for example. Is it possible to only train the read-out weights for the 3D trajectory?

All the neurons receive the teaching current and we do not have hidden neurons: we are considering the case of "fully observed network". Hidden neurons might be included by using the approaches proposed in [Brea2013] and [Rezende2014]. In these cases, the training on visible neurons is supervised (equivalent to ours) while the training for the hidden neurons is similar to a rewarded learning.

Yes, it is possible to only train the readout weights but the MSE would be higher. The result of this procedure is reported in the first bar of Fig.1C.

-It is claimed that spike-timing is a crucial feature of the presented model. Please, explain how the tasks (3D trajectories, walking dynamics and XOR) are related to exact spike timing in the RNN. Especially since the read-out seems to work based on filtered spike trains from the RNN.

The readout does not critically depend on the timing, even though the task is temporal and the temporal position of the spikes is relevant.

However, in order to solve the task, a specific sequence of spikes is learned (s_targ). This sequence has to be entirely and correctly recalled in order to successfully solve the task. This makes the timing of the spikes critical. In other words, the solution of the task is encoded in a specific pattern of spikes s_targ.

As shown in the panel below (now reported in the new Fig2), when the network is perturbed some of the spikes change their position in time (the error sum_it(abs(s_it-s_targ_it)) for sigma > 0.025, blue line) and the network is no longer able to reproduce the pattern s_targ. At the same time, the error on the task performance increases (the MSE significantly increases for sigma_noise^2/sigma_clock^2 > 0.01, red line). This means that recalling the correct spike timing and sequence was fundamental for correctly performing the task.

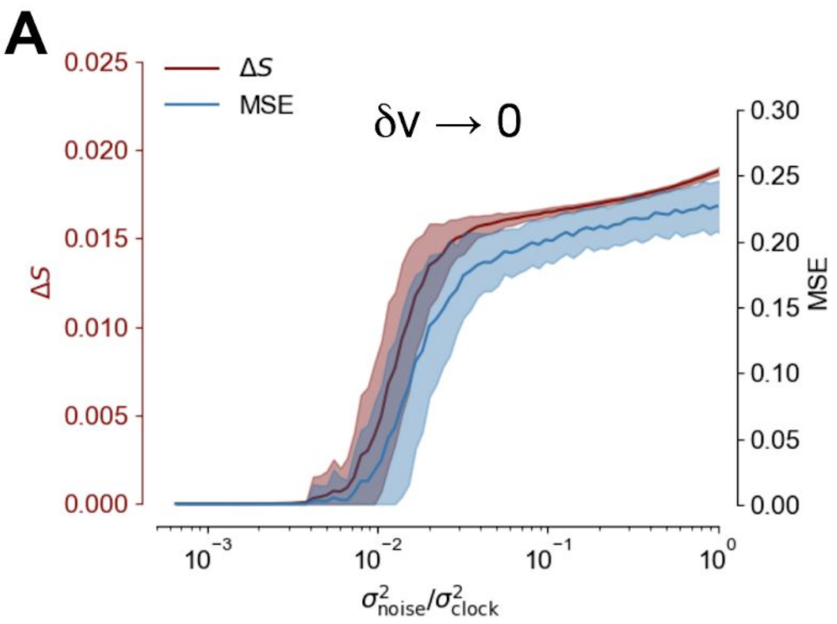

- At the end of the discussion the authors state: 'A major difference is that our learning method relies on optimizing the timing of the spikes emitted by each neuron rather that optimizing the time course of their input current.'

- As mentioned the algorithm is very similar to full-FORCE. Perhaps a more detailed analysis of similarities and differences would be useful?

We reply to both these points as follows:

In our model, we optimize the weights in order to reproduce a specific pattern of spikes (s_targ).

A target spiking pattern is not directly implied in the full-FORCE, where the total input current received by each from other neurons is considered as a target function. In the full-FORCE the weights are optimized to satisfy this constraint.

A first comment is that with this model it is not directly possible to impose a specific spatio-temporal pattern of spikes as a target, while coding with specific sequences of spikes is a relevant feature in biological neural networks as discussed in the **Introduction**.

A second comment is on the biological plausibility of a learning rule based on the input current. Indeed, in recent years, more and more use has been made of spike driven plasticity such as STDP.

Finally, we propose solutions that further improve the biological plausibility of the model, such as the online approximation and the online evaluation of target sequence within the neuron.

We expanded the sentence of the text mentioned by the reviewer:

“However, in our model, the weights are optimized in order to reproduce a specific pattern of spikes, a relevant feature in biological neural networks (see Introduction).

A target spatio-temporal spike pattern is not directly implied in the full-FORCE, where the total input current received by each from other neurons is considered as a target function.

Furthermore, we propose solutions that further improve the biological plausibility of the model, such as the online approximation and the online evaluation of the target sequence within the neuron."

-Is the model robust to perturbations?

We gauged the robustness to the noise by injecting Gaussian noise with 0 mean and variance sigma_nosie on top of the clock current. We recorded the retrieval MSE on the output trajectory as a function of the noise to signal ratio sigma_noise^2 / sigma_clock^2. We accumulated statistics over 50 experiments for each value of the noise amplitude sigma_noise.

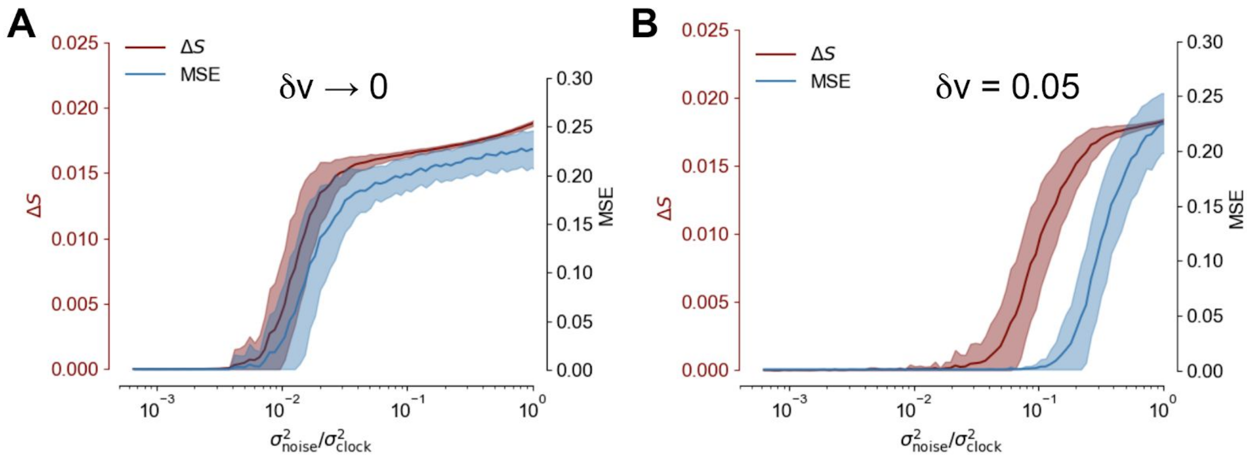

First tested our model with the standard rule we reported in eq (5) of the manuscript. We observed very weak robustness to noise since even a small perturbation induced a high MSE ((panel A, blue line)).

This led us to try a small variant of such a rule. We propose to take a finite dv in dJ = (s_targ-f(v,du)) dV/dJ (e.g. dv = 0.05).

When a finite dv is taken (dv=0.05) we observe remarkable stability to noise (panel B, blue line).

The advantage of the new rule proposed is the following.

When dv->0, it happens that if the target is S_targ=1, and v is just above the threshold (v>0), the update (s_targ-f(v,du)) = 0. A small amount of noise can bring v below the threshold and change the prediction of the network. If du>0, (s_targ-f(v,du)) > 0 is always positive, bringing the neuron further from the threshold. This will make the network much more robust to noise.

Moreover, the dv parameter can modulate the tolerance to failed spikes or to spikes with the wrong timing.

In the case dv->0 (panel A), when the network is perturbed some of the spikes change their position in time and the network is no longer able to reproduce the pattern s_targ. This effect can be seen in panel A, where the red line is an estimation of the number of spikes not following

the target pattern (Delta D = 1/NT sum_it(abs(s_it-s_targ_it))). At the same time the error on the task performance increases (the MSE significantly increases for sigma_noise^2/sigma_clock^2 > 0.01, panel A, blue line).

For dv= 0.05, (panel B) the system becomes much more robust to perturbations. Under moderate noise, the timing of the spike is perturbed (e.g. for sigma_noise^2/sigma_clock^2 = 0.1, panel B, red line) however the network is still able to perform the task (low MSE, e.g. for sigma_noise^2/sigma_clock^2 = 0.1, blue line). This means that the system became tolerant to the perturbation of the position of a certain number of spikes.

Finally, we comment that the first rule (where the limit dv->0 is taken) resembles the standard stdp (as already discussed in the main text), while the second one (a finite dv) is coherent with the plasticity rule proposed in [Clopath2010], which explicitly depends on the membrane potential of the postsynaptic neuron.

We included such information in the new figure (Fig2) and in the new section **Robustness to noise**.

-The cartoon of the model is not readable, maybe due to the pdf conversion of the figure.

We thank the reviewer for the comment, we improved the readability of the figure.

-Line 137: what is K? What does the clock input look like? A figure would help here.

We show below a picture showing how the clock is built (for the K=5 case). First, we build the clock signal, which is a KxT matrix with a thick diagonal structure (each row possessing 1/K-th of non-zero entries, ones, across the diagonal). The clock current is obtained by projecting this signal into the network with a random gaussian projection matrix (zero mean and custom variance) of shape NxK (not shown here), thus producing a final matrix of shape NxT which is used as the Input Clock Current.

We included a sketch of the clock signal in Fig1.

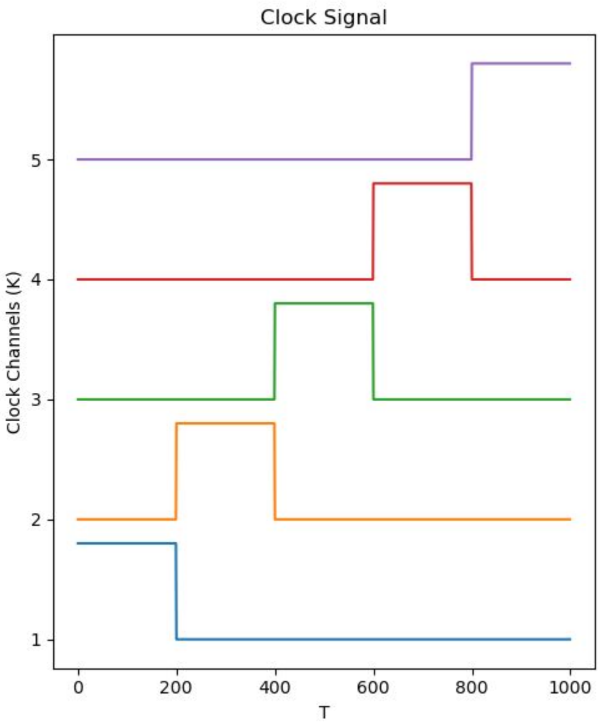

- Line 267: other neuron models, can you show an implementation? Maybe a figure comparing performance of the two different neuron models?

We implemented the coBa neuron and the respective learning rule.

In the coBa the excitatory and the inhibitory synapses have to be defined at the beginning because they have different ways to influence the postsynaptic neuron. This can be done by segregating excitatory and inhibitory neurons. For this reason, in our comparison, both the cuBa and the cuBa network has a predefined separation between excitatory and inhibitory neurons.

It follows the result of our comparison on the task of learning a 3D trajectory (the parameters are the same as in figure 1 except, time = 0.1s, sigma train = 1). The thin lines represent the spike error DS = 1/NT sum_it(abs(s_targ-s_pred)) for the single experiment, while the thick line is the average over 10 experiments. coBa and cuBa performances are reported in black and red respectively. The performances of the two networks are comparable in terms of error convergence.

We reported such a result in the **Supporting information**.

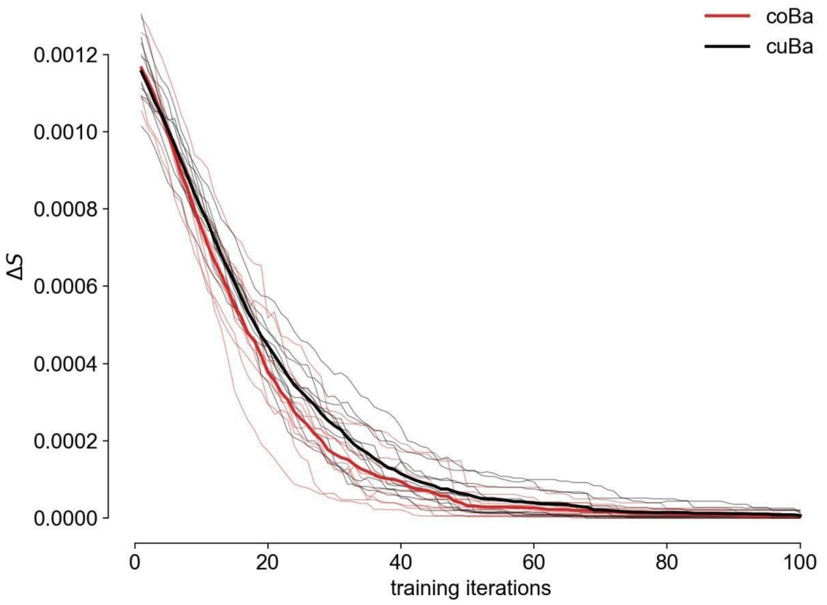

Some statements are made without explaining them, or citing literature.

Line 80: ‘makes the learning extremely faster’ why?

We observe a very efficient convergence, after only “100” steps the mse obtained is extremely low (on average below 0.02).

An error-based approach, (e.g. e-prop) iteratively proceeds optimizing the internal sequence of spikes minimizing progressively minimizing the MSE. In our model, we directly define a suited s_targ sequence. The learning is facilitated because the network is trained from the beginning to reproduce the desired internal sequence.

This sequence is strongly correlated to the target output, making more efficient the training of the readout.

We included the following text in the manuscript (Abstract):

“While error-based approaches, (e.g. e-prop) trial after trial optimize the internal sequence of spikes in order to progressively minimize the MSE we assume that a signal randomly projected from an external origin (e.g. from other brain areas) directly defines the target sequence. This facilitates the learning procedure since the network is trained from the beginning to reproduce *the desired internal sequence.”*

Line 178: clock is not necessary, show this? Why?

A target sequence can be learned and recalled also without a temporally structured input (what we call clock). The target sequence is reproduced only by recurrent connections. In this case, the sequence is initiated by providing the first spikes of the sequence (e.g. by providing pulse input currents to the network).

We actually considered the case with a clock to be completely comparable to previous benchmarks [Bellec2019, Nicola2018].

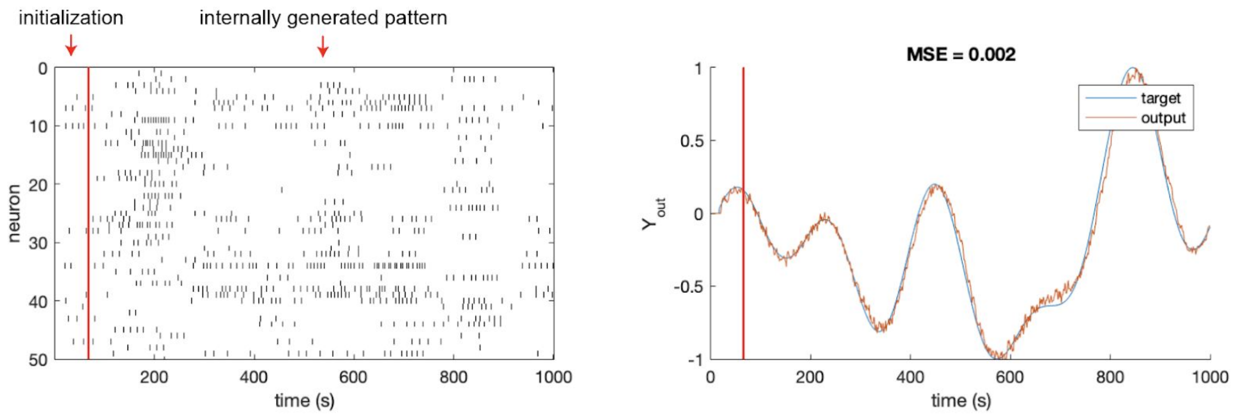

This results is now reported in the **Supporting informations**

Line 197: reference?

In reference [24] it is explained the acquisition of the data and its basic properties.

Line 330: where does this N^2 x T x P come from?

The computational time is proportional to the number of synapses and to the number of repetitions. The learning signals are evaluated on the whole time of the trial (indeed, there is a sum over time the expression of the learning rule) and that's why it is also proportional to the time.

We added the following sentence in the text:

"Indeed, the computational time is proportional to the number of synapses, to the trial duration and to the number of repetitions."

Line 424: reference? Why is the membrane potential related to the learning speed?

This is an empirical observation, which we did not report in the manuscript. To support such a statement we show the average convergence of the MSE, while learning the 3D trajectory for 3 different values of the time scale of the membrane potential 8ms, 4ms, and 2ms with the same learning rate eta = 0.125 (respectively in blue, red and yellow).

We reported such results in the **Supporting information**.

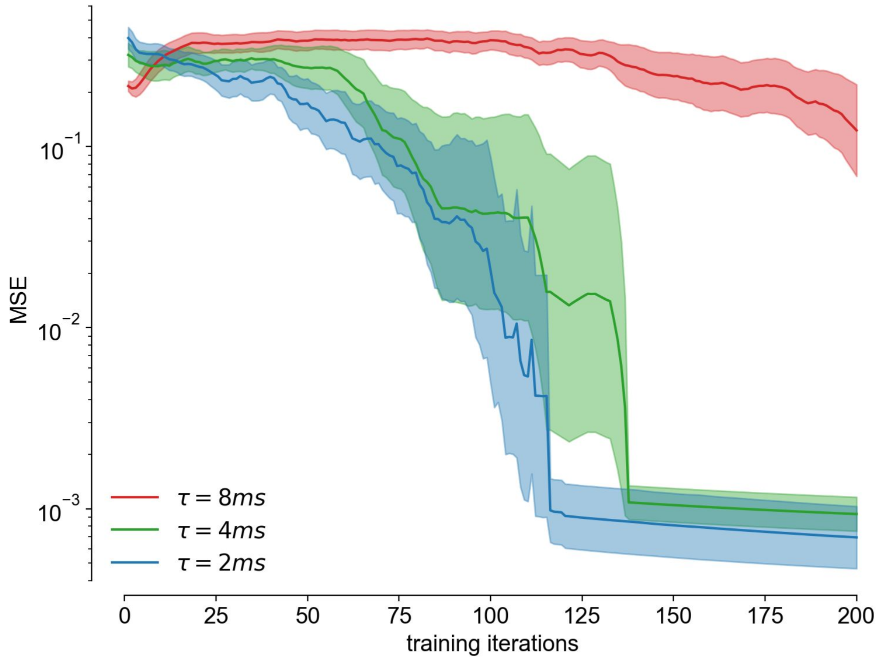

Table 1: Why is the membrane potential for the online approximation four times smaller than the other tasks (2ms vs 8ms)?

If indeed this is related to learning speed it could explain why the online version of the learning rule is faster.

We always compare online approximation and the simple gradient with the same timescale. In the comparison presented in Fig4, both the networks have tau_m = 2ms and tau_s=1.25ms. We included a new sentence to remark this in the main text (line 327):

“We compared the performances of the gradient ascent and its online approximation on this version of the task.”

To further support the claim that the superiority of the online approximation is not a consequence of the smaller timescale used for the experiment, we repeated the same online - simple gradient comparison for the task described in Fig1 (learning a 3D trajectory, T = 1000, tau_m = 8ms, tau_s=2ms).

Results are presented in the figure below: the online rule is again shown to be faster than the gradient ascent.

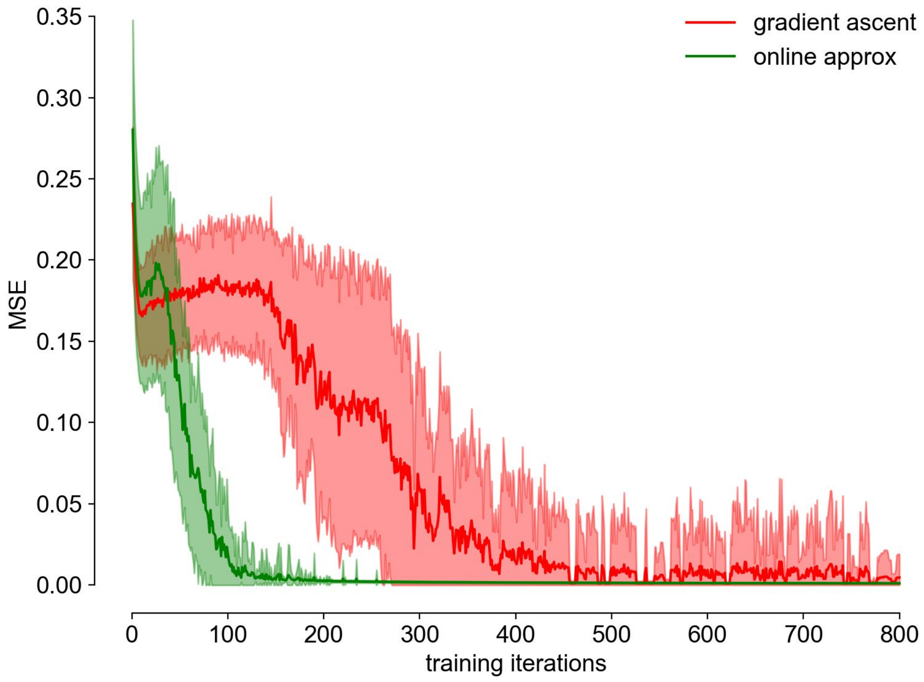

FIGURE: Test of online vs. gradient training. Model parameter: N=500, T=1000, tau_m=8ms, tau_s=2ms, dv=0.05, eta=10.

Could you make the code available for the reviewers?

The source code is available for download under a CC-BY license in the https://github.com/myscience/LTTS repository. Please cite this paper in derived works.

Eq. (1): Left-hand side does not depend on v, right-hand side does. The same problem appears in (13) and (14). It would be beneficial for the reader to explain this here.

This is correct. We specified this in the text (line 150):

"\newtext{In the left-hand side of eq(1) the dependence on $\bm{v}^{t}$ is not explicit. This is because $\bm{v}^{t}$ depends on $\bm{s}^{t}$, and is uniquely defined by setting a specific initial condition $\bm{v}^t= \bm{v}^0$ and the following rule:}"

Check the time indices in (16).

Yes thanks, we fixed it.

113; "We refer to such likelihood as L(s_targ ; J) where J are the recurrent synaptic weights of the system": (log)-likelihood was already introduced in (1), so this is a bit confusing.

That's true, we fixed it.

188; What are "peculiar rotational degrees of freedom"?

The term was used improperly. We removed it.

Some claims should be supported by references, e.g., before equation (7) (is it a novel equation, or is it based on the literature?), "the human brain works with only 20 watt".

We added the following sentence in the text, which includes a new reference (line 5):

"To justify the search for biological learning principles it is enough to consider that the human brain works with \newtext{a baseline power consumption estimated at about 13 watt, of which 75% spent on spike generation and transmission [Mckenna 2011]."

Are neurons or synapses conductance- or current-based? Why are connections segregated into excitatory and inhibitory in the case of coBa, but not cuBa? Why no in-silico experiments were performed with coBa?

Conductance based neurons are differently affected by excitatory and inhibitory synapses (in a voltage-dependent fashion). For example, when the membrane potential increases, the effect of excitation is depressed while the effect of inhibition is potentiated. For this reason, it is usually convenient to separate excitatory and inhibitory neurons. Current-based neurons do not have such a distinction between excitation and inhibition.

We performed experiments with networks with coBa neurons, obtaining performances comparable to the ones obtained with cuBa neurons. We note that in this comparison the segregation between excitatory and inhibitory has been implemented also in cuBa networks.

It follows the result of our comparison on the task of learning a 3D trajectory (the parameters are the same as in figure 1 except, time = 0.1s, sigma train = 1). The thin lines represent the spike error DS = sum_it(abs(s_targ-s_pred)) for the single experiment, while the thick line is the average over 10 experiments. coBa and cuBa performances are reported in black and red respectively. The performances of the two networks are comparable in terms of error convergence.

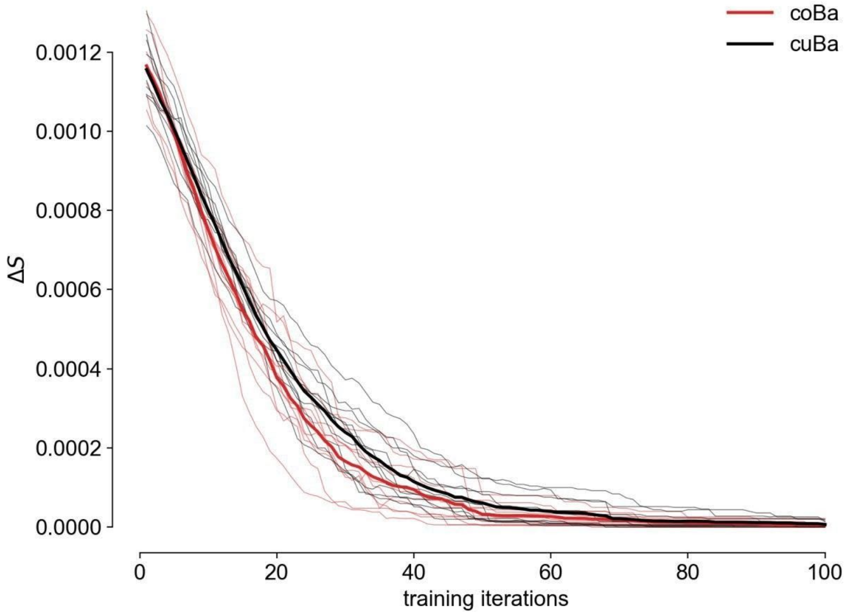

We included such a result in the **Supporting information**.

The "generation mode" is not very biologically plausible: the neural network is always set in the same initial condition. To be more realistic the network should start from random initial conditions and be cued by the input.

To test the robustness of the model to the initial condition we checked what happens when the system is initialized with a random initial condition on the membrane potential (v0 = -4 + N(0,sigma), where N(0,sigma) is a random variable with zero mean and variance sigma). We see that up to a large variation on such a value (sigma = 1) the retrieval is robust (the MSE is below 0.01). Note that sigma/(v_threshod - v_rest) = 0.25, the variance is large if compared to the distance between the resting and the threshold.

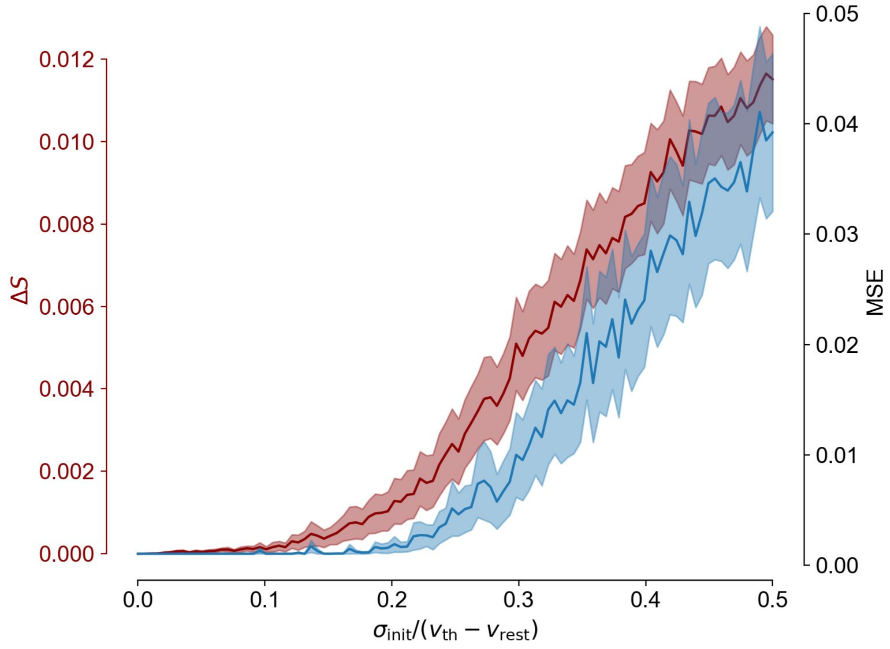

We included such a result in the **Supporting information**.

It is not clear whether the reported performance results are typical, average (over many training instances) or cherry-picked.

They are typical. To support this claim we computed the average MSE and its STD after 1000 iterations over 50 realizations for the learning of the 3d trajectory shown in Fig1.B. We obtain the following results.

E-prop: (0.014 ± 0.005)

Our method:

dv -> 0: (0.001 ± 0.0003)
dv = 0.05: (0.0010 ± 0.0004)
v = 0.2: (0.0010 ± 0.0003)

This is now included in the text.

"Learning to generate 3D trajectories". In [Bellec et al, 2019] they use random amplitude drawn from [0.5, 2.0] range. Is 2.5 a typo here?

The conditions should be the same for correct method comparisons.

Yes, it is a typo, we are using the same parameters. We corrected it in the text.

Algorithm performance comparison. A confidence interval should be provided for the method's MSE (despite the fact that Bellect et al don't provide such data). The protocol can be run for different inputs to collect statistics.

We collected statistics to answer this question.

The average MSE and its STD after 1000 iterations is computed over 50 trials. We obtain the following results.

E-prop: (0.014 ± 0.005)

Our method:

Delta_v -> 0: (0.00098 ± 0.00026)
Delta_v = 0.05: (0.0010 ± 0.0004)
Delta_v = 0.2: (0.0010 ± 0.0003)

We included such information in the main text.

It would be nice to illustrate faster convergence of new protocol in a plot together with the data from Bellec et al.

We compare the convergences of the MSE.

As described above, we run 100 instances of our model's training (each time producing a novel target trajectory) with parameters N=500, T=1000, eta = 0.5 for online training mode of 500 epochs via simple Gradient Descent (Non è Ascent visto che risaliamo la likelihood?). The readout weights are trained with eta_ro = 0.00075. In figures below, we report the results for the mse (lines:average, shaded areas standard deviation).

E-prop (blue) is compared to our model for the voltage-dependent learning rule (red)..

We included such a comparison in the **Supporting information**.

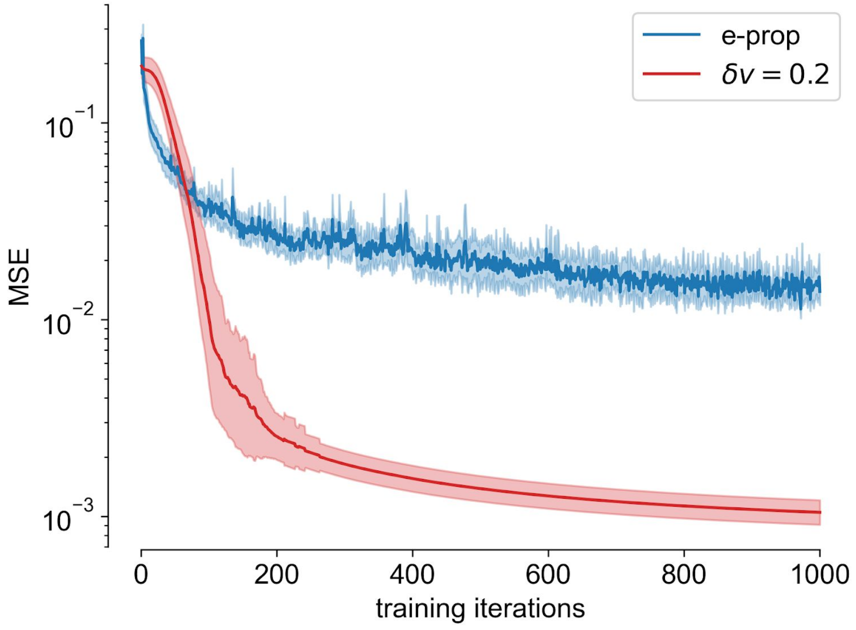

"Online approximation" is used only for "few representations" task ?

If so, it should be explained why it doesn't work well for the other tasks if it helps faster learning.

It works for all the tasks. We show the online vs gradient comparison for the 3D trajectory task shown in Fig1. The result suggests that the online is much faster also in this case.

This comparison is now included in the **Supporting information**.

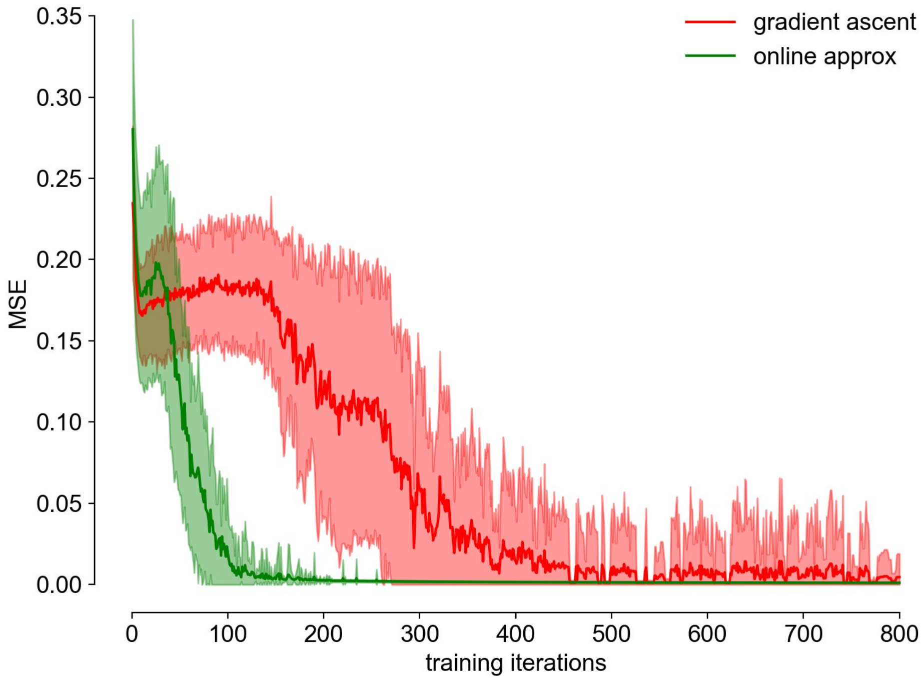

The citation for "Diederik P. Kingma and Jimmy Lei Ba. Adam : A method for stochastic optimization. 2014. arXiv:1412.6980v9" is needed.

We included this reference in the main text.

Spelling and grammatical errors:

We corrected the errors.

Abstract ...require very few examples.✓

Line 53: 'what is called' This sentence might benefit from a rewrite. ✓

Line 87: p(s(t)|v(t)) = .. spiking dynamics at time t is a function of membrane potential at time t, in equation 2 however it is the spiking dynamics at time t+1. Is this a mistake or something I missed? ✓

Line 108: ‘the spikes undergo’ ✓

53: what is call -> what is called ✓

74: vice versa ✓

108: spikes undergoes -> spikes undergo ✓

132: relationship ✓

Fig 1 caption :

... can be though -> can be thought ✓

... the different -> the difference ✓

... It is thus shown how our algorithm ... -> It is thus shown that our algorithm … ✓

164: turned off ✓

Fig 3 caption: Rastergam -> Rastergram ✓

Formula 6: the last symbol alignment ✓

271: the learning rule ✓

276: distinguishes ✓

282: requires paying attention ✓

293: the learning ✓

337: is inspire (?) ✓

340: used to derive (?) ✓

341: double [20] citation ✓

344: full-FORCE ✓

346: on the other hand ✓

349: a misplaced citation - suggests[25] ✓

Comments to the Author

1. Is the manuscript technically sound, and do the data support the conclusions?

The manuscript must describe a technically sound piece of scientific research with data that supports the conclusions. Experiments must have been conducted rigorously, with appropriate

controls, replication, and sample sizes. The conclusions must be drawn appropriately based on the data presented.

Reviewer #1: Partly

Reviewer #2: Partly

Reviewer #3: Yes

2. Has the statistical analysis been performed appropriately and rigorously?

Reviewer #1: N/A

Reviewer #2: I Don't Know

Reviewer #3: No

3. Have the authors made all data underlying the findings in their manuscript fully available?

The PLOS Data policy requires authors to make all data underlying the findings described in their manuscript fully available without restriction, with rare exception (please refer to the Data Availability Statement in the manuscript PDF file). The data should be provided as part of the manuscript or its supporting information, or deposited to a public repository. For example, in addition to summary statistics, the data points behind means, medians and variance measures should be available. If there are restrictions on publicly sharing data—e.g. participant privacy or use of data from a third party—those must be specified.

Reviewer #1: No

Reviewer #2: No

Reviewer #3: No

4. Is the manuscript presented in an intelligible fashion and written in standard English?

PLOS ONE does not copyedit accepted manuscripts, so the language in submitted articles must be clear, correct, and unambiguous. Any typographical or grammatical errors should be corrected at revision, so please note any specific errors here.

Reviewer #1: Yes

Reviewer #2: No

Reviewer #3: Yes

5. Review Comments to the Author

Please use the space provided to explain your answers to the questions above. You may also include additional comments for the author, including concerns about dual publication, research

ethics, or publication ethics. (Please upload your review as an attachment if it exceeds 20,000 characters)

Reviewer #1

Reviewer #1: The manuscript titled ‘Target spiking patterns enable efficient and biologically plausible learning for complex temporal tasks’ by Muratore et al. contains an interesting proposal. By providing a target signal to the neurons in an RNN they derive a learning rule that changes the parameters of the network to match the target signal. Most interestingly they show that the non-trivial temporal xor task can be learned. While potentially relevant for the study of spiking networks and temporal behaviour, some points have to be clarified and extended before publication can be recommended. Below I summarize the main points.

1. Novelty

-The study of Brea et al. (Journal of neuroscience 2013) seems very closely related to this study. In fact, the derivation of the learning rule for the specific dynamics is almost the same. It would be helpful to discuss the differences/advances made in this study.

Brea et al. assume that learning in the recurrent network consists of adapting all the synaptic weights such that the KL divergence between the target activity and the generated one is as small as possible.

Our approach is similar since we adjust for the recurrent weights by maximizing the probability of the network to generate a specific spatio-temporal spiking pattern.

Brea et al mainly focused on producing a target pattern of spikes (similarly to [Pfister 2006], [Rezende 2014], [Gardner 2015]). Here we have shown the possibility to use the generation of an internal sequence of spikes to solve complex and real life tasks. One example is the store and recall of a walking pattern.

Also, we introduce the use of realistic models of neurons, such as cuBa (current-based) and coBa (conductance-based) Leaky Integrate and Fire neurons.

Finally, we demonstrated the remarkable advantage to use the online version of the learning rule when the learning rate is large.

We added this reference in the introduction (line 45):

“Similarly, others \cite{brea2013matching} assumed that learning in the recurrent network consists of adapting all the synaptic weights such that the Kullback-Leibler divergence between the target activity and the generated one is as small as possible.”

-Fast and accurate learning of tasks has been proposed recently in various models, for example in Klos et al. (arXiv 2019) and Nicola and Clopath (Nat neuro 2019). It would be helpful to understand how this work relates to theirs.

Klos et al. (arXiv 2019) propose a recurrent network which dynamically adapts (not requiring synaptic plasticity) to reproduce a desired generic sequence belonging to a class of sequences, after a suitable pre-training.

The model is extremely interesting and flexible. However, the authors consider a network composed of rate neurons. They do not face the problems related to learning with spikes (e.g. see [Bellec 2020]) and it is not evident whether the same approach could be extended to recurrent spiking networks.

Nicola and Clopath (Nat neuro 2019) focus on reproducing several aspects of learning and replay in the thalamus (fast learning, compression, and inversion of sequences) leveraging RLS algorithms (FORCE algorithm).

The authors store a sequence by training the readout weights, from the hippocampal (SHOT-CA3) layer to the cortical one (RO-CA1). The hippocampal layer was pre-trained through FORCE algorithm to express an ordered sequence of oscillations.

The readout is learned with what the authors call the Fourier rule. This rule memorizes a target trajectory by learning a set of decoders, which use the presynaptic oscillatory activity as a temporal basis to reconstruct the trajectory. Moreover, the basis has to be orthogonal, otherwise, the learning rule would be non-local and biologically implausible.

Unlike this model, we train simultaneously the recurrent and the readout weights. The recurrent networks are trained to learn a task-specific internal coding (and not a generic spatio-temporal pattern used for all the tasks). This choice allows us to get a very high precision in the learned sequences (we achieve very low MSE), an aspect that Nicola and Clopath do not seem to investigate.

Finally, we remark that we show how we are able to solve complex temporal tasks (the temporal xor) and store and recall high dimensional dynamics (walking dynamics).

In both the models mentioned by the reviewer, fast learning relies on a suited preparation or pre-training of the network. This is an aspect we do not investigate.

Our focus is to make learning more efficient by proposing a task-specific internal coding and through the online approximation.

We included the references to these papers in the main text (line 448):

"Models for fast learning have been proposed, and in general they rely on a suited pre-training or preparation of the recurrent network \cite{Bellec2019,nicola2019diversity,klos2020dynamical,maes2020learning}. This procedure is not task specific. Subsequently, the training on the task is performed. We do not investigate an optimization of the network before the learning task. However, we find that our time local approximation is extremely advantageous when learning from a small number of examples, increasing significantly the learning velocity."

- Some models based on realistic plasticity (stdp-like) and dynamics have recently been proposed to learn storage and recall of sequences. A few examples are Murray and Escola (eLife 2017), Maes et al. (plos cb 2020), Cone and Shouval (arXiv 2020). To what extent are the ideas in these papers similar? For example also in these models a target input is provided and learned (in a few different ways).

Murray and Escola (eLife 2017) present a model where the speed of the learned trajectory can be modulated by the amplitude of the tonic input to the network.

The sequences to be learned are induced by a structured input (similarly to our work) and weights are shaped by anti-Hebbian plasticity (anti-Hebbian stdp in the spiking model). This is because the network is composed of all inhibitory neurons.

Similarly, we induce an integral target through external stimulation, however, our learning rule is not merely Hebbian. Our plasticity rule includes depression if a spike is emitted when the target is not to spike.

Maes et al. (plos cb 2020) propose a model where a spiking recurrent network of excitatory and inhibitory is trained to encode time which is then mapped through the read-out neurons to encode an arbitrary trajectory.

Similarly to (Murray and Escola**)** the target dynamics of the recurrent spiking network is induced by external input and it is learned by voltage-dependent stpd.

This approach is, in some sense, similar to what proposed in (Nicola and Clopath)**.** In the first stage, a generic spatio-temporal pattern is induced in the recurrent network. In a second stage, the readout is trained from this temporal basis to the desired output.

As we remarked above, the internal dynamics induced in our recurrent network is task-specific, unlike what (Maes et al.) propose, allowing for higher precision in the desired output.

We included this citation in the main text together with (Nicola and Clopath) at line 454**.**

Cone and Shouval (arXiv 2020) propose a model which, combining modular structure with a learning rule based on eligibility traces, can accurately learn and recall sequences of up to at least 8 elements having different positions and durations.

Here the authors provide a specific solution to a specific problem. We do not directly face this kind of problem, however in [Rezende2014], where a formalism similar to ours is used, a correlated task is considered. The task consists in storing and recalling a stair pattern, a sequence of states of different durations. The authors claim that to achieve it, it is necessary to include hidden units in the recurrent network (see Fig.3A in [Rezende2014]).

We included the reference to Cone and Shouval (arXiv 2020) in the manuscript together with other target-based approaches. (line 31)

“A first approach to implement a target based training consists in inducing in the recurrent network, through an external stimulus, a spatio-temporal pattern of activity, which is learned by

the network through Hebbian plasticity \cite{murray2017learning,cone2020learning, capone2019sleep}."

All these models learn a target internal dynamics, induced by a temporally structured external input, through Hebbian synaptic plasticity rule (e.g. stdp).

In certain conditions, our rule, which results from likelihood maximization, is compatible with stdp (as discussed in [Pfister2006]).
However, our learning rule is not merely Hebbian. It indeed takes into account the target spike and the prediction of the network. When the target is to spike but the neuron doesn't, the synapses are potentiated, while when the target is not to spike and the prediction is to spike, it is depressed.

Finally, we remark that the target dynamics in the referred works are the firing rate patterns of the neurons (or populations of neurons), while our target is a specific pattern of spikes. The possibility to achieve this is of great biological relevance, as discussed in the **Introduction**. Also, we believe that this allows us to get a very low error on the target output (e.g. the 3D trajectory).

2. Training

-It is not clear to me how the 'internal target spike trains' are obtained. I can see that there is a teaching input current I_teach present, but how are the spike trains obtained from this? I assume that they are recorded during spontaneous dynamics and afterwards in a training phase are used as a target where the I_teach is not present anymore.

This is correct, the target pattern of spikes is the pattern induced by I_teach in the network in absence of recurrent synapses. In the end, the learning of the recurrent synapsis should allow the network to recall the spiking target in absence of I_teach.

We clarified such point in the manuscript (line 107):

"We define the internal target $s_{i, \mathrm{targ}}^t$ as the pattern of spikes induced in the network by the training current $\bm{I}_\mathrm{teach}^t$, in absence of recurrent connections, possibly in combination with an additional input signal that, depending on the task, can be either superfluous or essential. In the memory recall tasks the latter may serve as an external clock, in others (e.g. XOR task) it is the input to be processed to solve the task."

-The teaching current is projected randomly to all the neurons in the network. What if some neurons do not receive a teaching current? In Brea et al. there is a pool of hidden neurons for example. Is it possible to only train the read-out weights for the 3D trajectory?

All the neurons receive the teaching current and we do not have hidden neurons: we are considering the case of "fully observed network". Hidden neurons might be included by using the approaches proposed in [Brea2013] and [Rezende2014]. In these cases, the training on visible neurons is supervised (equivalent to ours) while the training for the hidden neurons is similar to a rewarded learning.

Yes, it is possible to only train the readout weights but the MSE would be higher. The result of this procedure is reported in the first bar of Fig.1C.

-It is claimed that spike-timing is a crucial feature of the presented model. However, I do not understand how the tasks (3D trajectories, walking dynamics and XOR) are related to exact spike timing in the RNN. Especially since the read-out seems to work based on filtered spike trains from the RNN.

The readout does not critically depend on the timing, even though the task is temporal and the temporal position of the spikes is relevant.

However, in order to solve the task, a specific sequence of spikes is learned (s_targ). This sequence has to be entirely and correctly recalled in order to successfully solve the task. This makes the timing of the spikes critical. In other words, the solution of the task is encoded in a specific pattern of spikes s_targ.

As shown in the panel below (now reported in the new Fig2), when the network is perturbed some of the spikes change their position in time (the error sum_it(abs(s_it-s_targ_it)) for sigma > 0.025, blue line) and the network is no longer able to reproduce the pattern s_targ. At the same time, the error on the task performance increases (the MSE significantly increases for sigma_noise^2/sigma_clock^2 > 0.01, red line). This means that recalling the correct spike timing and sequence was fundamental for correctly performing the task.

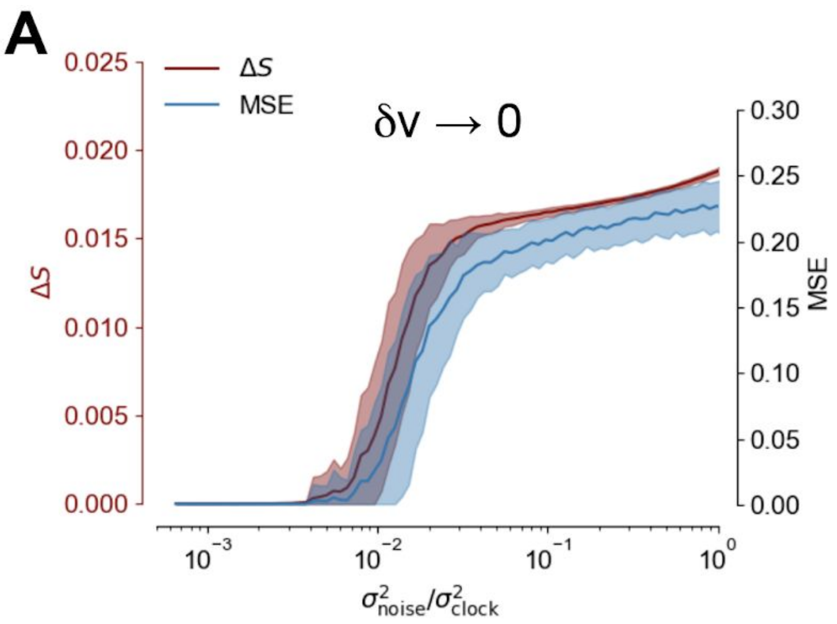

-At the end of the discussion the authors state: 'A major difference is that our learning method relies on optimizing the timing of the spikes emitted by each neuron rather that optimizing the time course of their input current.' My question here is similar to what I wrote above: I am not sure how this works.

In our model, we optimize the weights in order to reproduce a specific pattern of spikes (s_targ).

A target spiking pattern is not directly implied in the full-FORCE, where the total input current received by each from other neurons is considered as a target function. In the full-FORCE the weights are optimized to satisfy this constraint.

A first comment is that with this model it is not directly possible to impose a specific spatio-temporal pattern of spikes as a target, while coding with specific sequences of spikes is a relevant feature in biological neural networks as discussed in the **Introduction**.

A second comment is on the biological plausibility of a learning rule based on the input current. Indeed, in recent years, more and more use has been made of spike driven plasticity such as STDP.

Finally, we propose solutions that further improve the biological plausibility of the model, such as the online approximation and the online evaluation of target sequence within the neuron.

We expanded the sentence of the text mentioned by the reviewer:

“However, in our model, the weights are optimized in order to reproduce a specific pattern of spikes, a relevant feature in biological neural networks (see Introduction).

A target spatio-temporal spike pattern is not directly implied in the full-FORCE, where the total input current received by each from other neurons is considered as a target function.

Furthermore, we propose solutions that further improve the biological plausibility of the model, such as the online approximation and the online evaluation of the target sequence within the neuron.”

-Is the model robust to perturbations?

We gauged the robustness to the noise by injecting Gaussian noise with 0 mean and variance sigma_nosie on top of the clock current. We recorded the retrieval MSE on the output trajectory as a function of the noise to signal ratio sigma_noise^2 / sigma_clock^2. We accumulated statistics over 50 experiments for each value of the noise amplitude sigma_noise.

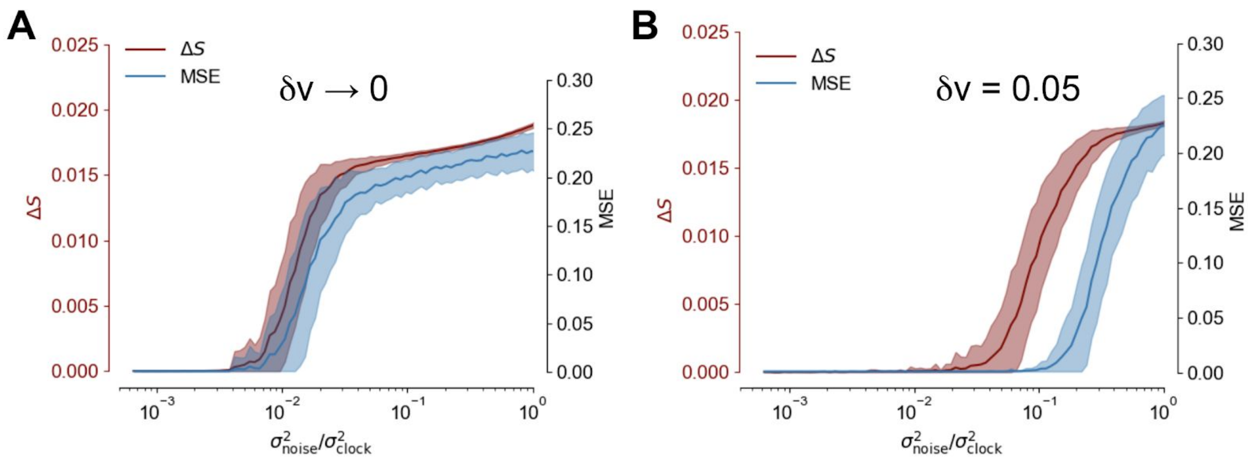

First tested our model with the standard rule we reported in eq (5) of the manuscript. We observed very weak robustness to noise since even a small perturbation induced a high MSE ((panel A, blue line)).

This led us to try a small variant of such a rule. We propose to take a finite dv in dJ = (s_targ-f(v,du)) dV/dJ (e.g. dv = 0.05).

When a finite dv is taken (dv=0.05) we observe remarkable stability to noise (panel B, blue line).

The advantage of the new rule proposed is the following.

When dv->0, it happens that if the target is S_targ=1, and v is just above the threshold (v>0), the update (s_targ-f(v,du)) = 0. A small amount of noise can bring v below the threshold and change the prediction of the network. If du>0, (s_targ-f(v,du)) > 0 is always positive, bringing the neuron further from the threshold. This will make the network much more robust to noise.

Moreover, the dv parameter can modulate the tolerance to failed spikes or to spikes with the wrong timing.

In the case dv->0 (panel A), when the network is perturbed some of the spikes change their position in time and the network is no longer able to reproduce the pattern s_targ. This effect can be seen in panel A, where the red line is an estimation of the number of spikes not following the target pattern (Delta D = 1/NT sum_it(abs(s_it-s_targ_it))). At the same time the error on the task performance increases (the MSE significantly increases for sigma_noise^2/sigma_clock^2 > 0.01, panel A, blue line).

For dv= 0.05, (panel B) the system becomes much more robust to perturbations. Under moderate noise, the timing of the spike is perturbed (e.g. for sigma_noise^2/sigma_clock^2 = 0.1, panel B, red line) however the network is still able to perform the task (low MSE, e.g. for sigma_noise^2/sigma_clock^2 = 0.1, blue line). This means that the system became tolerant to the perturbation of the position of a certain number of spikes.

Finally, we comment that the first rule (where the limit dv->0 is taken) resembles the standard stdp (as already discussed in the main text), while the second one (a finite dv) is coherent with the plasticity rule proposed in [Clopath2010], which explicitly depends on the membrane potential of the postsynaptic neuron.

We included such information in the new figure (Fig2) and in the new section **Robustness to noise**.

3. Graphical presentation of results

-The cartoon of the model is not readable, maybe due to the pdf conversion of the figure.

We thank the reviewer for the comment, we improved the readability of the figure.

-Line 137: what is K? What does the clock input look like? A figure would help here.

We show below a picture showing how the clock is built (for the K=5 case). First, we build the clock signal, which is a KxT matrix with a thick diagonal structure (each row possessing 1/K-th of non-zero entries, ones, across the diagonal). The clock current is obtained by projecting this signal into the network with a random gaussian projection matrix (zero mean and custom variance) of shape NxK (not shown here), thus producing a final matrix of shape NxT which is used as the Input Clock Current.

We included a sketch of the clock signal in Fig1.

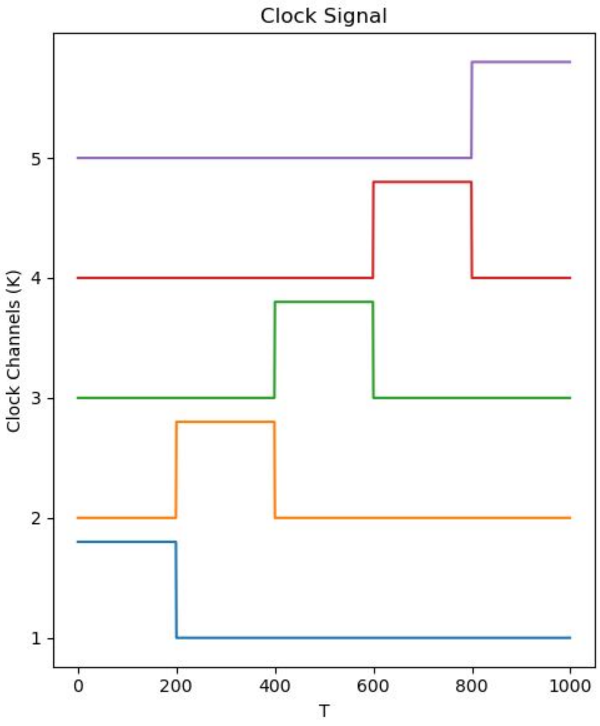

-Line 267: other neuron models, can you show an implementation? Maybe a figure comparing performance of the two different neuron models?

We implemented the coBa neuron and the respective learning rule.

In the coBa the excitatory and the inhibitory synapses have to be defined at the beginning because they have different ways to influence the postsynaptic neuron. This can be done by segregating excitatory and inhibitory neurons. For this reason, in our comparison, both the cuBa and the cuBa network has a predefined separation between excitatory and inhibitory neurons.

It follows the result of our comparison on the task of learning a 3D trajectory (the parameters are the same as in figure 1 except, time = 0.1s, sigma train = 1). The thin lines represent the spike error DS = sum_it(abs(s_targ-s_pred)) for the single experiment, while the thick line is the average over 10 experiments. coBa and cuBa performances are reported in black and red respectively. The performances of the two networks are comparable in terms of error convergence.

We reported such a result in the **Supporting information**.

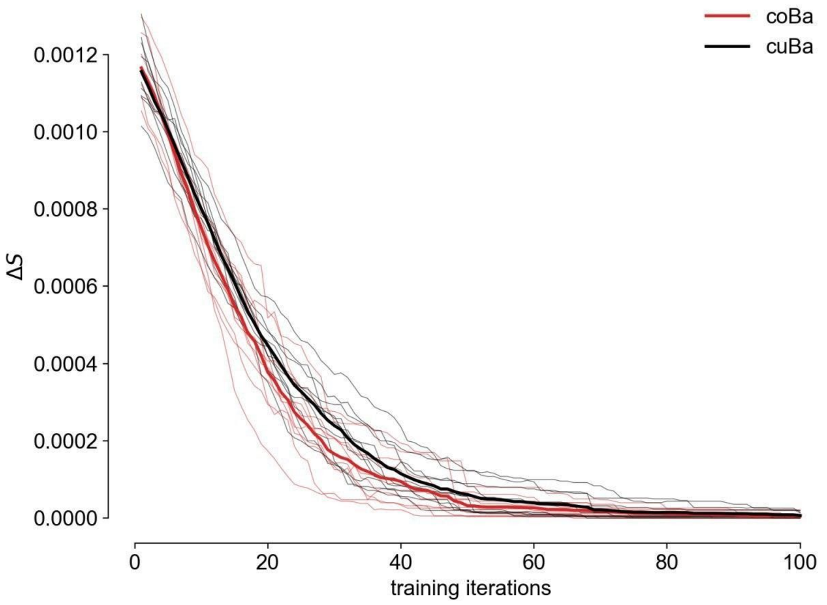

4. Explanations

Some statements are made without explaining them, or citing literature.

Line 80: ‘makes the learning extremely faster’ why?

We observe a very efficient convergence, after only “100” steps the mse obtained is extremely low (on average below 0.02).

An error-based approach, (e.g. e-prop) iteratively proceeds optimizing the internal sequence of spikes minimizing progressively minimizing the MSE. In our model, we directly define a suited s_targ sequence. The learning is facilitated because the network is trained from the beginning to reproduce the desired internal sequence.

This sequence is strongly correlated to the target output, making more efficient the training of the readout.

We included the following text in the manuscript (Abstract):

“While error-based approaches, (e.g. e-prop) trial after trial optimize the internal sequence of spikes in order to progressively minimize the MSE we assume that a signal randomly projected from an external origin (e.g. from other brain areas) directly defines the target sequence. This facilitates the learning procedure since the network is trained from the beginning to reproduce *the desired internal sequence.”*

Line 178: clock is not necessary, show this? Why?

A target sequence can be learned and recalled also without a temporally structured input (what we call clock). The target sequence is reproduced only by recurrent connections. In this case,

the sequence is initiated by providing the first spikes of the sequence (e.g. by providing pulse input currents to the network).

We actually considered the case with a clock to be completely comparable to previous benchmarks [Bellec2019, Nicola2018].

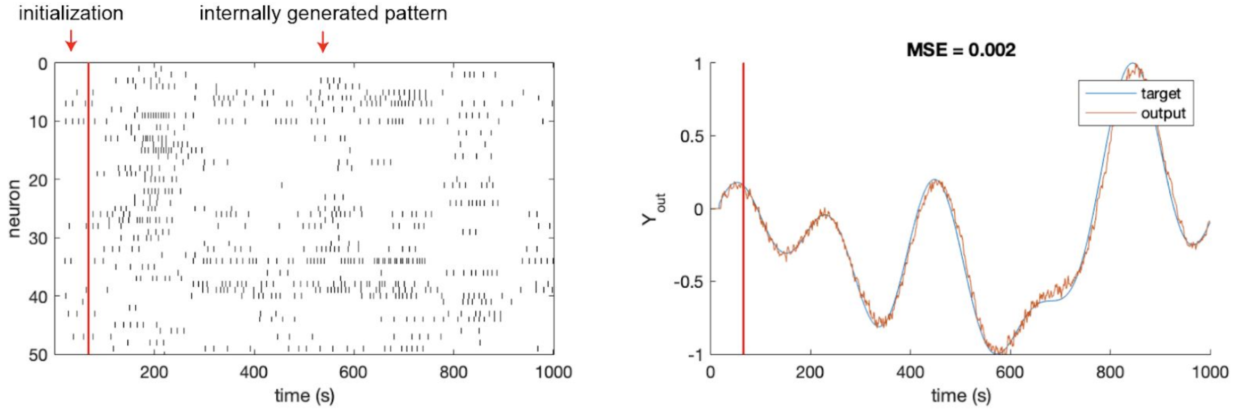

We included such a result in the **Supporting information**.

Line 197: reference?

In reference [24] it is explained the acquisition of the data and its basic properties.

Line 330: where does this N^2 x T x P come from?

The computational time is proportional to the number of synapses and to the number of repetitions. The learning signals are evaluated on the whole time of the trial (indeed, there is a sum over time the expression of the learning rule) and that's why it is also proportional to the time.

We added the following sentence in the text:

"Indeed, the computational time is proportional to the number of synapses, to the trial duration and to the number of repetitions."

Line 424: reference? Why is the membrane potential related to the learning speed?

This is an empirical observation, which we did not report in the manuscript. To support such a statement we show the average convergence of the MSE, while learning the 3D trajectory for 3 different values of the time scale of the membrane potential 8ms, 4ms, and 2ms with the same learning rate eta = 0.125 (respectively in blue, red and yellow).

We reported such results in the **Supporting information**.

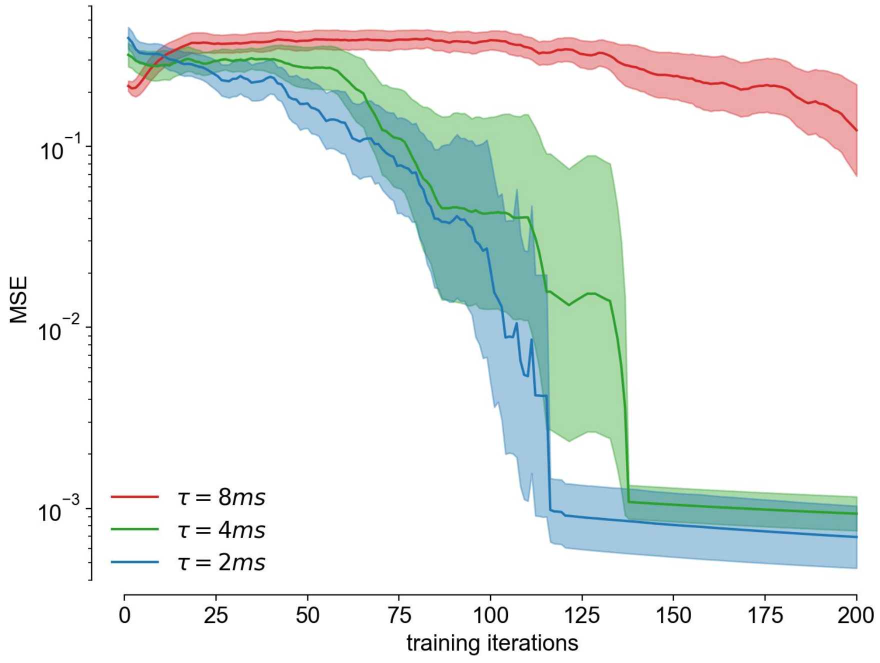

Table 1: Why is the membrane potential for the online approximation four times smaller than the other tasks (2ms vs 8ms)?

If indeed this is related to learning speed it could explain why the online version of the learning rule is faster.

We always compare online approximation and the simple gradient with the same timescale. In the comparison presented in Fig4, both the networks have tau_m = 2ms and tau_s=1.25ms. We included a new sentence to remark this in the main text (line 327):

“We compared the performances of the gradient ascent and its online approximation on this version of the task.”

To further support the claim that the superiority of the online approximation is not a consequence of the smaller timescale used for the experiment, we repeated the same online - simple gradient comparison for the task described in Fig1 (learning a 3D trajectory, T = 1000, tau_m = 8ms, tau_s=2ms).

Results are presented in the figure below: the online rule is again shown to be faster than the gradient ascent.

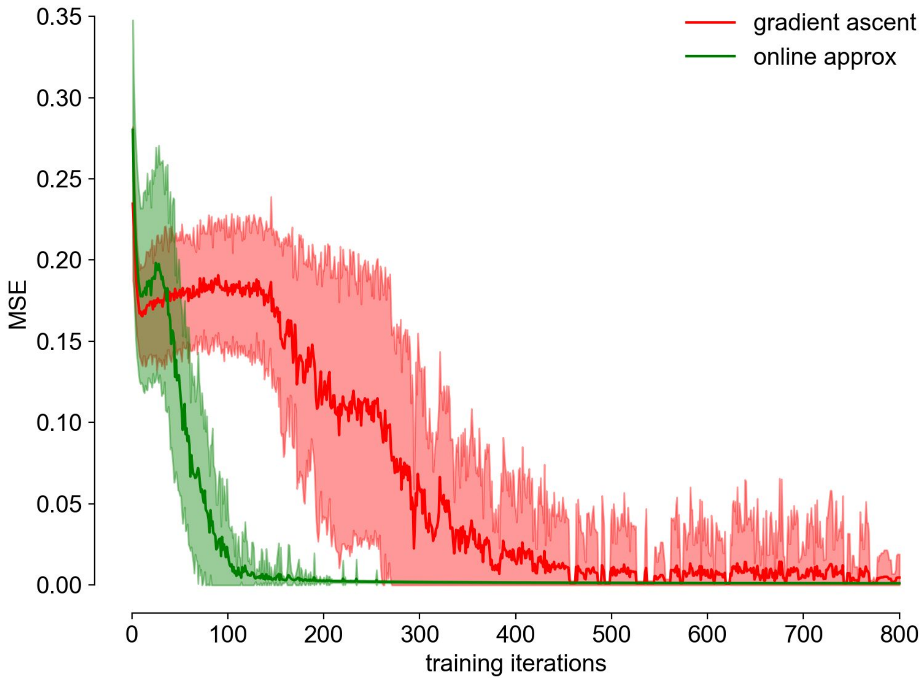

FIGURE: Test of online vs. gradient training. Model parameter: N=500, T=1000, tau_m=8ms, tau_s=2ms, dv=0.05, eta=10.

5. Language errors

Throughout the manuscript there are both spelling and grammatical errors. A few of those are listed here:

Abstract ...require very few examples. ✓

Line 53: ‘what is called’ This sentence might benefit from a rewrite. ✓

Line 87: p(s(t)|v(t)) = .. spiking dynamics at time t is a function of membrane potential at time t, in equation 2 however it is the spiking dynamics at time t+1. Is this a mistake or something I missed?

It was a typo, we corrected it.

Line 108: ‘the spikes undergo’ ✓

We corrected the errors.

6. Code

Is it possible that the code is made available for the reviewers?

The source code is available for download under a CC-BY license in the https://github.com/myscience/LTTS repository. Please cite this paper in derived works.

References for Reviewer#1

[Pfister2006] Pfister JP, Toyoizumi T, Barber D, Gerstner W. Optimal spike-timing-dependent plasticity for precise action potential firing in supervised learning. Neural computation. 2006;18(6):1318{1348.
[Rezende 2014] Jimenez Rezende D, Gerstner W. Stochastic variational learning in recurrent spiking networks. Frontiers in Computational Neuroscience. 2014;8:38. doi:10.3389/fncom.2014.00038.

[Gardner2015] Gardner B, Gruning A. Supervised learning in spiking neural networks for precise temporal encoding. PloS one. 2016;11(8):e0161335.

[Clopath2010] Clopath C, Busing L, Vasilaki E, Gerstner W. Connectivity reflects coding: a model of voltage-based STDP with homeostasis. Nature neuroscience. 2010;13(3):344.

[Nicola2017] Nicola W, Clopath C. Supervised learning in spiking neural networks with FORCE training. Nature communications. 2017;8(1):2208.

[Bellec2019] Bellec G, Scherr F, Hajek E, Salaj D, Legenstein R, Maass W. Biologically inspired alternatives to backpropagation through time for learning in recurrent neural nets. 2019; p. 1{37.

[Urbanczik2014] Urbanczik R, Senn W. Learning by the dendritic prediction of somatic spiking. Neuron. 2014;81(3):521{528.

[Mckenna2011] McKenna MC, Gruetter R, Sonnewald U, et al. Energy Methabolism of the Brain. In: Brady S, Siegel G, Albers RW, Price D, editors. Basic Neurochemistry. Principles of Molecular, Cellular and Medical Neurobiology. Elsevier; 2011. p. 557.

Reviewer #2

Reviewer #2: The manuscript introduces a simple training procedure for spiking neural networks based on two main ingredients: (a) outputs of the network are generated via trainable linear readout, and (b) dynamics of the network depends on the connectivity matrix, which is trained to generate target spike sequence by means of the standard maximization of the likelihood. Target activities are generated during a separate phase in which neurons are driven by inputs and fixed random linear feedback that encodes the desired outputs. The algorithm is tested in-silico on multiple tasks that show its efficiency.

Important issues:

The Authors claim to use leaky integrate and fire neurons (see Eq. (3)), but in the discussion no reset of v_t is mentioned. Such resets, while being a potentially essential source of non-linearity in the dynamics of the network, could be problematic when calculating derivatives of v_t with respect to J. Unfortunately this issue is not discussed.

After the spike, a negative term (- J_res * s_ii^t) affects the neuron provoking the after spike reset of the membrane potential. We mistakenly did not include it in the text. We corrected the equation including such a term (see new eq(4) and (13)).

Such formalization does not have derivability issues. The derivative of v_t with respect to the weights is defined iteratively (see new eq(18)). The reset induced by the spikes doesn't depend on J and doesn't appear in the expression of V_t/ dJ.

The Authors claim that their method is biologically plausible. However, equation (16) is recursive and requires the storage of the full history of membrane potentials and spikes throughout the training. This does not seem to be biologically plausible (two phases needed, and how are the values stored in the biological substrate?) and may be impractical in engineering applications. It is not clear to me whether the online version of the algorithm given by (6) alleviates these problems, because not enough details of this procedure are currently given. For example, are targets still generated during a separate phase in the online training paradigm?

Eq(16) (new eq(18)) only depends on the previous time step and no storage is required.

Indeed, in the simple gradient version of the learning, at every time dV_t/dJ can be evaluated from the previous time step, and a partial update DJ_t = (S_targ-S_pre) dV_t/dJ is evaluated. The partial updates for each time are accumulated until the end of the trial, after which the weights are eventually updated. This only requires the accumulation of partial updates.

The online approximation consists in performing online each partial update. This makes the accumulation not necessary.

Another point is the storing of the target pattern s_targ. Indeed, we evaluate it in a first phase and store it. This is true both for the simple gradient and the online approximation.

However, it is possible (and absolutely equivalent) to evaluate online this pattern at every trial. Indeed, the target pattern is the one generated by the teaching signal in absence of recurrent synaptic connection. This might be evaluated online by a dedicated compartment of the neuron that receives connections from the teacher and doesn't receive recurrent connections.

We claim this to be biologically plausible: in pyramidal neurons, it is known that the apical and basal compartment receive respectively contextual (or teaching) and sensory inputs [Larkum2013].

We explain better this point in the text:

"In terms of biological plausibility, we can assume that the target pattern of spikes is computed by a dedicated compartment of the neuron that receives the training current.

This would be totally equivalent to our protocol. It can be locally computed at every trial of the training procedure, making always accessible the target sequence to the network, without the requirement of an expensive storing of the target. We consider this assumption biologically plausible because of the increasing consensus on the specific role of the apical compartments of pyramidal neurons [Larkum2013] which receives contextual signals from other areas."

In order to properly place this work in the context of current research it would be beneficial for the reader to list other target-based learning schemes, e.g., [1] where targets are provided via auto-encoders, [2] where targets are generated by a chaotic neural network. Although in the manuscript the same network is first used to generate the target activity and then being trained, the target activity is fixed during training (i.e., second phase, see the discussion after (16)). If I understand correctly, recurrent connections are not used in the first phase. One could thus consider the target-generating network as distinct from the trained network, similarly to [2]. Another related work is [3], where it is shown that backpropagation-like algorithm works even if the feedback matrix is random and fixed. Since in the current work target activities are generated via random and fixed linear feedback, this seems relevant. Unfortunately, although this work is cited, this analogy is not discussed. Moreover, such a random feedback was used to train recurrent neural networks in [4]. Another related work is [5].

We thank the reviewer for very interesting references, we included them in the text:

Line 37

“Interestingly, target-based approaches have also been proposed for feedforward networks, where targets, which propagate instead of errors, can be assigned to each layer of the deep network [1].”

Line 34

“Other approaches propose to leverage the intrinsic dynamic of the network by imposing one chaotic sequence produced by the network as an internal target. This strategy has been explored for both rate [2] and spiking neurons [5].”

Line 22

“An important step towards biological plausibility has been to show that a backpropagation-like algorithm works even if the feedback matrix is random and fixed [3]. A similar principle can be used to obtain a biologically plausible approximate of BPTT and train recurrent neural networks [4]”

The manuscript contains numerous language errors and in some places is hard to understand.

Minor issues:

The Authors claim that the Python code with simulations will be available on GitHub. Unfortunately, it does not seem to be available now, so I could not check the code.

The source code is available for download under a CC-BY license in the https://github.com/myscience/LTTS repository. Please cite this paper in derived works.

Eq. (1): Left-hand side does not depend on v, right-hand side does. The same problem appears in (13) and (14). It would be beneficial for the reader to explain this here. I am assuming that the explanation is related to the fact that v in turn depends on the history of s (assuming some specified initial condition of v).

This is correct. We specified this in the text (line 150):

“\newtext{In the left-hand side of eq(1) the dependence on $\bm{v}^{t}$ is not explicit. This is because $\bm{v}^{t}$ depends on $\bm{s}^{t}$, and is uniquely defined by setting a specific initial condition $\bm{v}^t= \bm{v}^0$ and the following rule:}”

There is probably a mistake in the time indices in (16).

Yes thanks, we fixed it.

113; "We refer to such likelihood as L(s_targ ; J) where J are the recurrent synaptic weights of the system": (log)-likelihood was already introduced in (1), so this is a bit confusing.

That’s true, we fixed it.

118; I see nothing peculiar about the expression (5). In fact, it looks rather simple, and follows the well known form of delta learning rule.

The form of the expression in eq(5) is indeed rather standard, and similar to what proposed in [Bellec2019, Depasquale2018, Clopath2010] and equivalent to [Rezende2014, Gruning 2015, Pfister2006, Urbanczik2014]

However here we derive the specific rule required for cuBa and coBa neurons. In particular, the *“spike response function”* dV/dJ for coBa neurons is rather unusual (see new eq(10) and eq(11)).

188; What are "peculiar rotational degrees of freedom"?

The term was used improperly. We removed it.

As mentioned the algorithm is very similar to full-FORCE. Perhaps a more detailed analysis of similarities and differences would be useful?

In our model, we optimize the weights in order to reproduce a specific pattern of spikes (s_targ).

A target spiking pattern is not directly implied in the full-FORCE, where the total input current received by each from other neurons is considered as a target function. In the full-FORCE the weights are optimized to satisfy this constraint.

A first comment is that with this model it is not directly possible to impose a specific target of spike, while coding with specific sequences of spikes is a relevant feature in biological neural networks as discussed in the **Introduction**.

A second comment is on the biological plausibility of a learning rule based on the input current. Indeed, in recent years, more and more use has been made of spike driven plasticity such as STDP.

Finally, we propose solutions that further improve the biological plausibility of the model, such as the online approximation and the online evaluation of target sequence during the training.

We expanded the sentence of the text mentioned by the reviewer:

"However, in our model, the weights are optimized in order to reproduce a specific pattern of spikes, a relevant feature in biological neural networks (see Introduction).

A target spatio-temporal spike pattern is not directly implied in the full-force, where the total input current received by each from other neurons is considered as a target function.

Furthermore, we propose solutions that further improve the biological plausibility of the model, such as the online approximation and the online evaluation of target sequence during the training.*"*

Some claims should be supported by references, e.g., before equation (7) (is it a novel equation, or is it based on the literature?), "the human brain works with only 20 watt".

We added the following sentence in the text, which includes a new reference (line 5):

"To justify the search for biological learning principles it is enough to consider that the human brain works with \newtext{a baseline power consumption estimated at about 13 watt, of which 75% spent on spike generation and transmission [Mckenna 2011]."

Are neurons or synapses conductance- or current-based? Why are connections segregated into excitatory and inhibitory in the case of coBa, but not cuBa? Why no in-silico experiments were performed with coBa?

Conductance based neurons are differently affected by excitatory and inhibitory synapses (in a voltage-dependent fashion). For example, when the membrane potential increases, the effect of excitation is depressed while the effect of inhibition is potentiated. For this reason, it is usually convenient to separate excitatory and inhibitory neurons. Current-based neurons do not have such a distinction between excitation and inhibition.

We performed experiments with networks with coBa neurons, obtaining performances comparable to the ones obtained with cuBa neurons. We note that in this comparison the segregation between excitatory and inhibitory has been implemented also in cuBa networks.

It follows the result of our comparison on the task of learning a 3D trajectory (the parameters are the same as in figure 1 except, time = 0.1s, sigma train = 1). The thin lines represent the spike error DS = sum_it(abs(s_targ-s_pred)) for the single experiment, while the thick line is the average over 10 experiments. coBa and cuBa performances are reported in black and red respectively. The performances of the two networks are comparable in terms of error convergence.

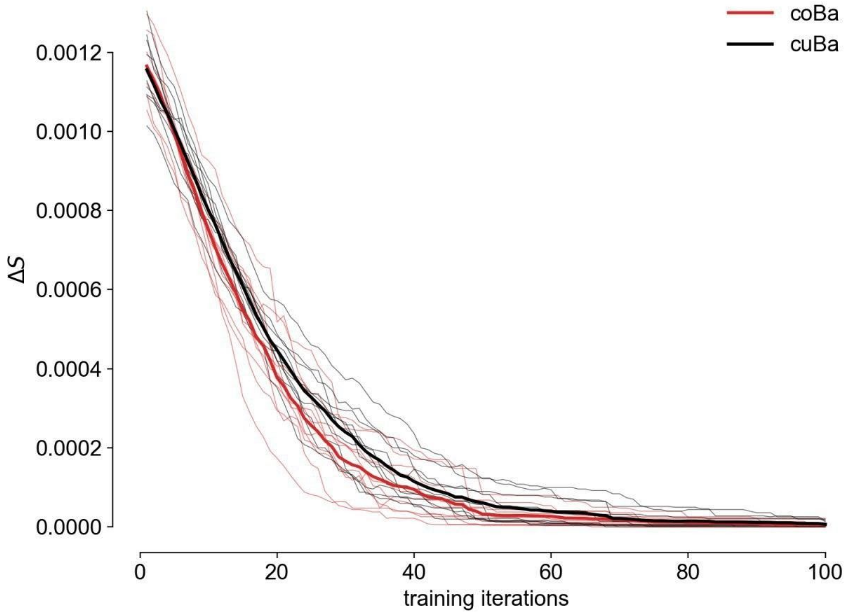

We included such a result in the **Supporting information**.

The "generation mode" is not very biologically plausible: the neural network is always set in the same initial condition. To be more realistic the network should start from random initial conditions and be cued by the input.

To test the robustness of the model to the initial condition we checked what happens when the system is initialized with a random initial condition on the membrane potential (v0 = -4 + N(0,sigma), where N(0,sigma) is a random variable with zero mean and variance sigma). We see that up to a large variation on such a value (sigma = 1) the retrieval is robust (the MSE is below 0.01). Note that sigma/(v_threshod - v_rest) = 0.25, the variance is large if compared to the distance between the resting and the threshold.

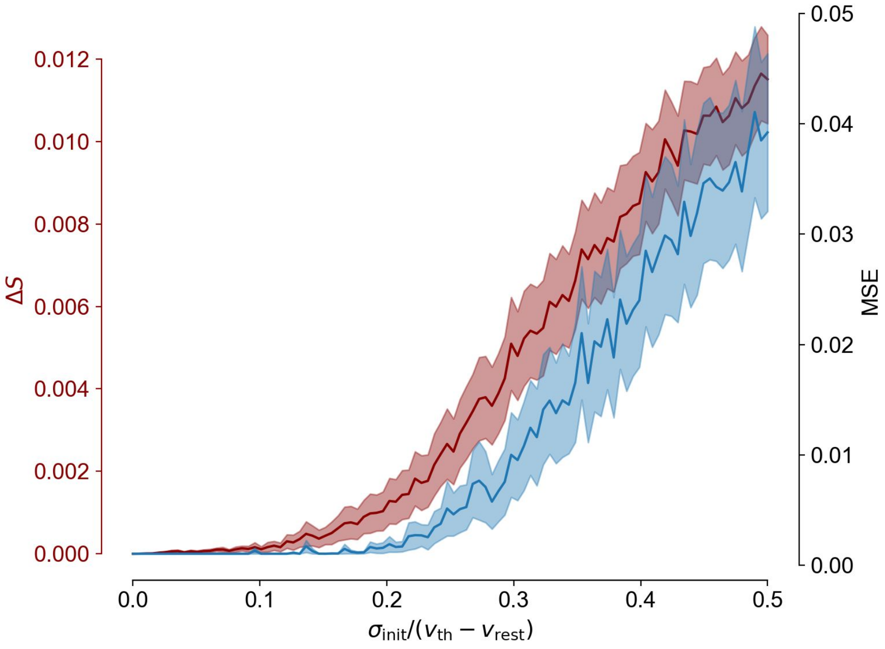

We included such a result in the **Supporting information**.

It is not clear whether the reported performance results are typical, average (over many training instances) or cherry-picked.

They are typical. To support this claim we computed the average MSE and its STD after 1000 iterations over 50 realizations for the learning of the 3d trajectory shown in Fig1.B. We obtain the following results.

E-prop: (0.014 ± 0.005)

Our method:

dv -> 0: (0.001 ± 0.0003)
dv = 0.05: (0.0010 ± 0.0004)
v = 0.2: (0.0010 ± 0.0003)

This is now included in the text.

[1] Dong-Hyun Lee, Saizheng Zhang, Asja Fischer and Yoshua Bengio, Difference Target Propagation, in Machine Learning and Knowledge Discovery in Databases, pages 498-515, Springer International Publishing, 2015

[2] Laje R, Buonomano DV. Robust timing and motor patterns by taming chaos in recurrent neural networks. Nat. Neurosci. 2013;16: 925–933. pmid:23708144

[3] Lillicrap, T., Cownden, D., Tweed, D. et al. Random synaptic feedback weights support error backpropagation for deep learning. Nat Commun 7, 13276 (2016)

[4] Murray, James M. Local online learning in recurrent networks with random feedback. eLife 8 (2019): e43299.

[5] Kim, Christopher M., and Carson C. Chow. Learning recurrent dynamics in spiking networks. eLife 7 (2018): e37124.

References for reviewer 2

[Larkum2013] Larkum ME. A cellular mechanism for cortical associations: an organizing principle for the cerebral cortex. Trends in Neurosciences. 2013;36:141-151.
[Bellec2019] Bellec G, Scherr F, Hajek E, Salaj D, Legenstein R, Maass W. Biologically inspired alternatives to backpropagation through time for learning in recurrent neural nets. 2019; p. 1-37.

[Depasquale2018] DePasquale B, Cueva CJ, Rajan K, Abbott L, et al. full-FORCE: A target-based method for training recurrent networks. PloS one. 2018;13(2):e0191527.

[Clopath2010] Clopath C, Busing L, Vasilaki E, Gerstner W. Connectivity reflects coding: a model of voltage-based STDP with homeostasis. Nature neuroscience. 2010;13(3):344.

[Rezende2014] Jimenez Rezende D, Gerstner W. Stochastic variational learning in recurrent spiking networks. Frontiers in Computational Neuroscience. 2014;8:38. doi:10.3389/fncom.2014.00038.

[Gruning2015] Gardner B, Gruning A. Supervised learning in spiking neural networks for precise temporal encoding. PloS one. 2016;11(8):e0161335.

[Pfister2006] Pfister JP, Toyoizumi T, Barber D, Gerstner W. Optimal spike-timing-dependent plasticity for precise action potential firing in supervised learning. Neural computation. 2006;18(6):1318{1348.

[Urbanczik2014] Urbanczik R, Senn W. Learning by the dendritic prediction of somatic spiking. Neuron. 2014;81(3):521{528.

[Mckenna2011] McKenna MC, Gruetter R, Sonnewald U, et al. Energy Methabolism of the Brain. In: Brady S, Siegel G, Albers RW, Price D, editors. Basic Neurochemistry. Principles of Molecular, Cellular and Medical Neurobiology. Elsevier; 2011. p. 557.

Reviewer #3

Reviewer #3: The authors propose a protocol for training recurrent spiking neural networks(RSNN) without using direct error back propagation.

The protocol uses targed-based learning approach and closely related to full-FORCE training method described in DePasquale et al, 2018.

The network is trained to recapitulate the activity induced during the "pre-training" phase, when the target signal is provided as the input.

The proposed approach is biologically plausible and provides interesting insights into possible mechanisms of biological neural networks learning. The references to 2 compartment neuron models are especially interesting.

According to my assessment, the authors need to address the minor points listed below.

1. "Learning to generate 3D trajectories". In [Bellec et al, 2019] they use random amplitude drawn from [0.5, 2.0] range. Is 2.5 a typo here?

The conditions should be the same for correct method comparisons.

Yes, it is a typo, we are using the same parameters. We corrected it in the text.

2. Algorithm performance comparison. A confidence interval should be provided for the method's MSE (despite the fact that Bellect et al don't provide such data).

The protocol can be run for different inputs to collect statistics.

We collected statistics to answer this question.

The average MSE and its STD after 1000 iterations is computed over 50 trials. We obtain the following results.

E-prop: (0.014 ± 0.005)

Our method:

Delta_v -> 0: (0.00098 ± 0.00026)

Delta_v = 0.05: (0.0010 ± 0.0004)

Delta_v = 0.2: (0.0010 ± 0.0003)

We included such information in the main text.

3. It would be nice to illustrate faster convergence of new protocol in a plot

together with the data from Bellec et al.

We compare the convergences of the MSE.

As described above, we run 100 instances of our model's training (each time producing a novel target trajectory) with parameters N=500, T=1000, eta = 0.5 for online training mode of 500 epochs via simple gradient ascent. The readout weights are trained with eta_ro = 0.00075. In figures below, we report the results for the mse (lines:average, shaded areas standard deviation).

E-prop (blue) is compared to our model for the voltage-dependent learning rule (red).

We included such a comparison in the **Supporting information**.

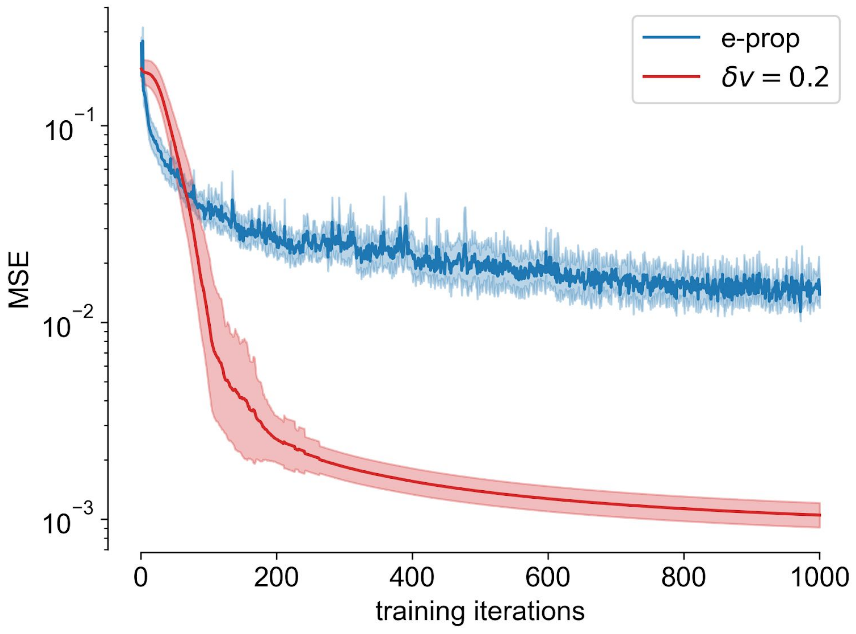

4. "Online approximation" is used only for "few representations" task?

If so, it should be explained why it doesn't work well for the other tasks if it helps faster learning.

It works for all the tasks. We show the online vs gradient comparison for the 3D trajectory task shown in Fig1. The result suggests that the online is much faster also in this case.

This comparison is now included in the **Supporting information**.

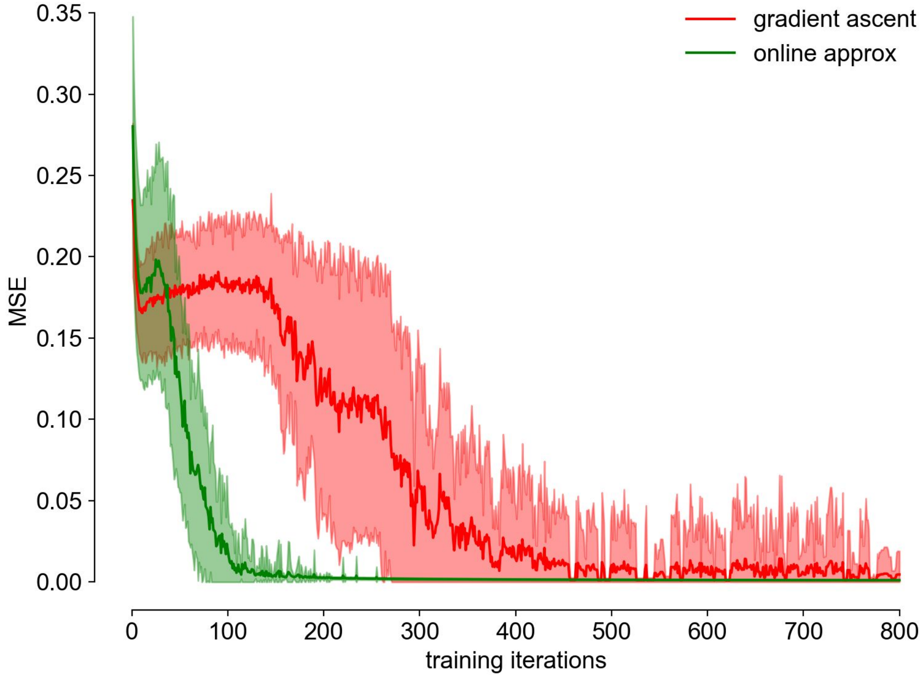

5. The citation for "Diederik P. Kingma and Jimmy Lei Ba. Adam : A method for stochastic optimization. 2014. arXiv:1412.6980v9" is needed.

We included this reference in the main text.

Typos

53: what is call -> what is called ✓

74: vice versa ✓

108: spikes undergoes -> spikes undergo ✓

132: relationship ✓

Fig 1 caption :

... can be though -> can be thought ✓

... the different -> the difference ✓

... It is thus shown how our algorithm ... -> It is thus shown that our algorithm … ✓

164: turned off ✓

Fig 3 caption:

Rastergam -> Rastergram ✓

Formula 6: the last symbol alignment ✓

271: the learning rule ✓

276: distinguishes ✓

282: requires paying attention ✓

293: the learning ✓

337: is inspire (?) ✓

340: used to derive (?) ✓

341: double [20] citation ✓

344: full-FORCE ✓

346: on the other hand ✓

349: a misplaced citation - suggests[25] ✓

We corrected the typos.
