# Supplementary material for: Target spike patterns enable efficient and biologically plausible learning for complex temporal tasks
Source: PLoS One. 2021 Feb 16;16(2):e0247014. doi: 10.1371/journal.pone.0247014 (PMC7886200; doi:10.1371/journal.pone.0247014)
Supplement: Attachment — Submitted filename: Reply to reviewers II - PONE2020.pdf [file pone.0247014.s004.pdf]

Reply to Reviewers - 2

Target spike patterns enable efficient and biologically plausible learning for complex temporal tasks

Paolo Muratore, Cristiano Capone, Pier Stanislao Paolucci

Editor 1

Reviewer 1 5

Reviewer 2 5

Reviewer 3 6

Editor

Dear Dr. Muratore,

Thank you for submitting your manuscript to PLOS ONE. After careful consideration, we feel that it has merit but does not fully meet PLOS ONE's publication criteria as it currently stands. Therefore, we invite you to submit a revised version of the manuscript that addresses the points raised during the review process.

Please, check whether during training s_pred and v at time t are calculated assuming that at times t_past?

-Is it possible to make a statement about the capacity of the model? For example, the temporal xor task shows that at least 4 different inputs can be stored in a network of 500 neurons, how many more can be stored?

-It would be nice to mention full-FORCE approach in the introduction in more explicit manner, since that is the most closely related algorithm published so far.

Not all of the source code is provided, e.g. coBa model is missing

- It is well-known that in the studied scenario minimizing KL divergence is equivalent to maximizing likelihood. Why do you contrast these two approaches?

-- "Statistics is evaluated over 50 realizations for each σ noise value. (25 different 3D trajectory times 25 realizations of the noisy input.)": This is confusing. 25 times 25 equals 50?

-- Equations (12), (13), and (14), (15) are exactly the same as, respectively, (5), (4) and (2), (1). Perhaps such a repetition is not needed?

Language:

-- "In addition, our approach results quite natural in terms of biological plausibility.": ungrammatical

-- "combined to a deterministic generation of the spikes": combined --> compared ?

-- "bringing the neuron further from the threshold and ensuring a safety gap": also called margin

line 205 parenthesis

line 312 "The in the case"

Reviewer 1

Reviewer #1: The manuscript has been improved greatly.

One question I would like to ask is whether it is possible to make a statement about the capacity of the model. For example, the temporal xor task shows that at least 4 different inputs can be stored in a network of 500 neurons, how many more can be stored?

To give an answer to this question in this task, we considered the more generic case of parity task, which is the natural extension of a cascade of XOR producing a single output bit for more than two input bits. These new results are now included in the **Supporting Information.**

The output should be positive if the number of 1 is odd, and negative if the number of 1 is even. A number of bits are presented one after the other. At the end the network has to produce his answer. We evaluated both the 3 and 4 bits input cases. These two cases correspond respectively to 8 and 16 possible temporal patterns.

When we try this for the 3 bit case, we obtain that the maximum number of correctly classified patterns is 6. See figure below.

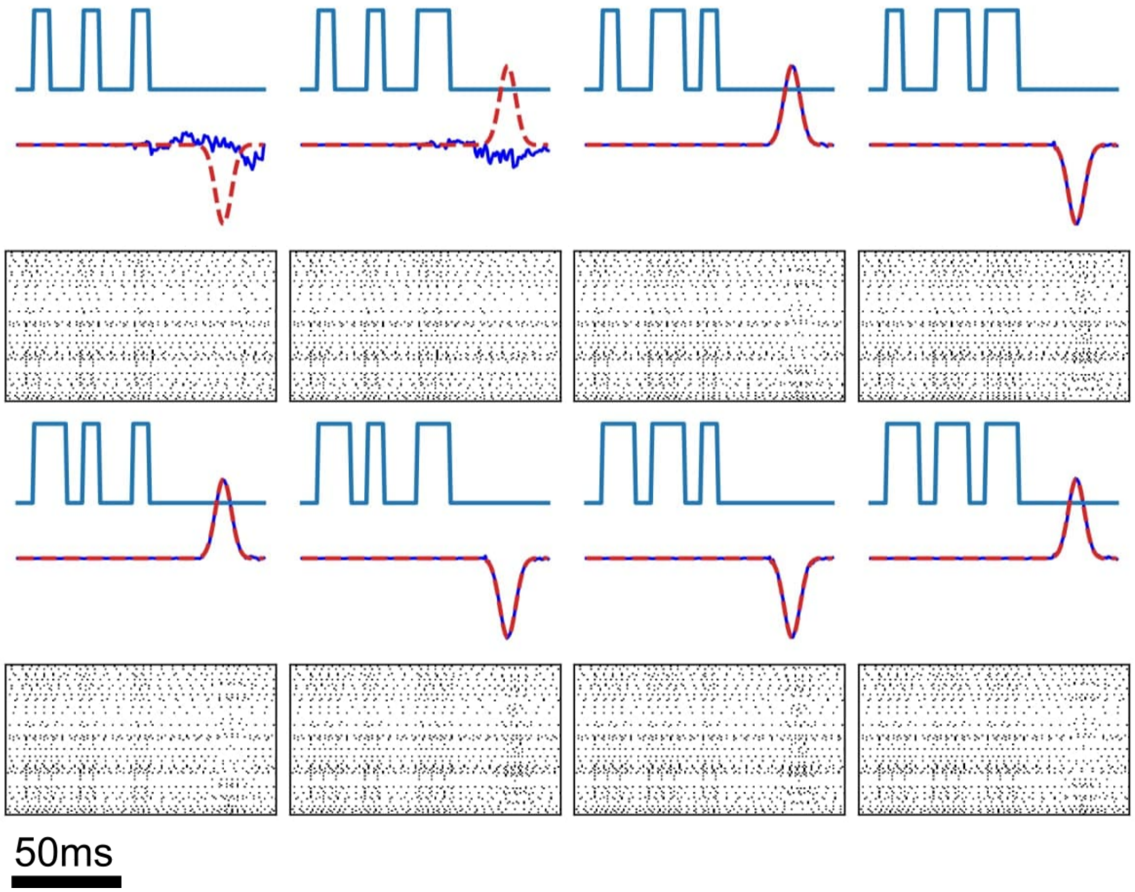

We tried to go further and we included a new feature in the model. Indeed, when the difficulty of temporal computation increases, it is difficult for the network to achieve very good results in absence of recurrent connections.

For this reason we introduced random recurrent weights between the apical compartments of the neurons. This only changes the way the target pattern of spikes is evaluated, which is indeed done under the influence of these recurrent apical connections.

Using this new definition of the target pattern of spikes s_targ we obtain better results and all the 8 patterns of the 3-bit-parity are correctly classified (see figure below).

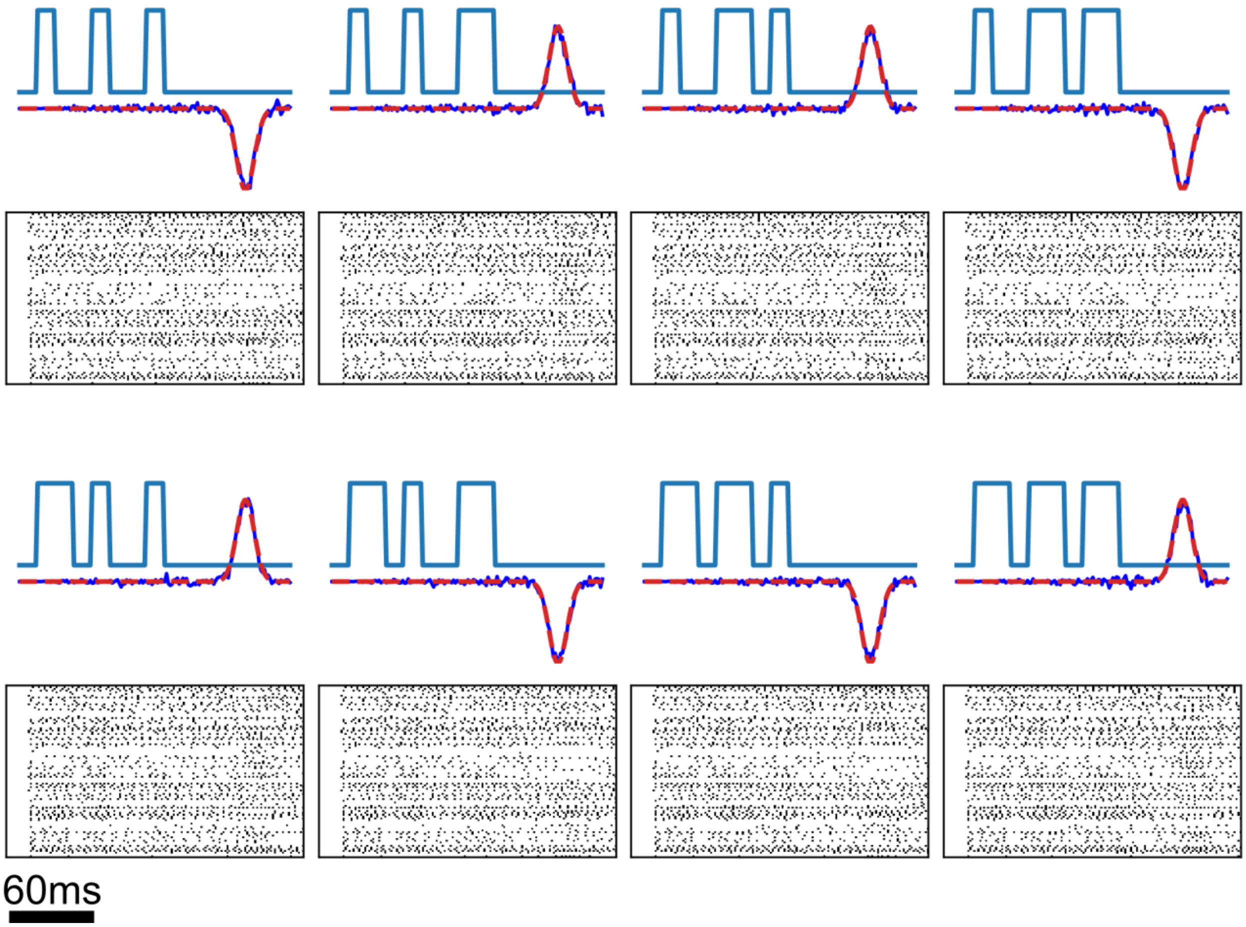

We also tried the 4-bit-parity, 14 out of the 16 patterns are correctly classified (see below).

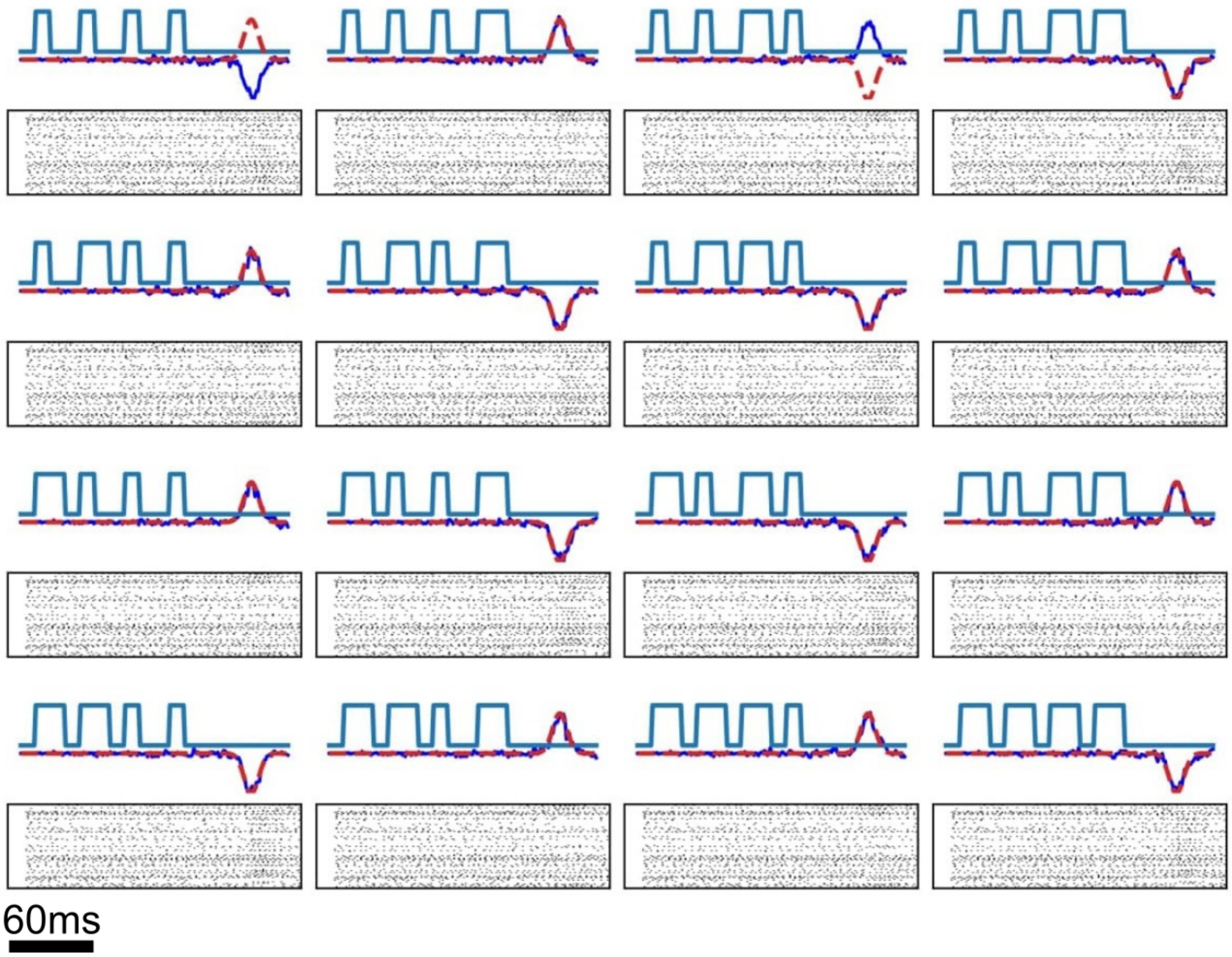

Reviewer 2

Reviewer #2: In my opinion the Authors have properly addressed most issues raised by me and other Referees. The code in Python is written clearly and is easily accessible through GitHub. Altogether the current version of the manuscript merits its publications in PLOS One. However, I found some other issues that should be addressed before the manuscript is published:

Potentially important:

-- If I understood correctly, during training s_pred and v at time t are calculated assuming that at times t_past

We apologize but we didn't understand this point. There is probably a typo in the sentence?

Minor:

-- It is well-known that in the studied scenario minimizing KL divergence is equivalent to maximizing likelihood. I am not sure why the Authors contrast these two approaches.

That's correct. Our intention was not to contrast them. To avoid this misunderstanding we rephrased the text as follows:

"Other papers have proposed probability based approaches assuming that learning in the recurrent network consists of adapting all the synaptic weights such that the Kullback-Leibler divergence between the target activity and the generated one is as small as possible (or similarly such that likelihood of the target activity is as large as possible) \cite{pfister2006optimal,brea2013matching,rezende2014stochastic,gardner2016supervised}. "

-- "Statistics is evaluated over 50 realizations for each σ noise value. (25 different 3D trajectory times 25 realizations of the noisy input.)": This is confusing. 25 times 25 equals 50?

Indeed, it was a typo, the total number of realizations is 25x25=625. We corrected the text. We thank the reviewer for the observation.

-- "Inspired by the stochastic nature of the single neuronal unit": As far as I know, in reality single-neuron dynamics is quite robustly deterministic. Synapses are known to be more noisy.

The noise described in the neuron accounts for many effects as synaptic noise and the effective noise coming from other areas of the brain.

In the **Discussion** section, we replaced the criticized sentence, with the following statement.

"*Even though biological neurons have quite deterministic dynamics, the modeling of additional noise is required to reproduce the effects of synaptic noise and of noisy signals coming from other areas of the brain. For this reason the single neuron can be approximated as a stochastic unit whose dynamics is described by a likelihood.*"

-- Equations (12), (13), and (14), (15) are exactly the same as, respectively, (5), (4) and (2), (1). Perhaps such a repetition is not needed?

This is true. We removed the numbers from the equations in the methods citing the number of their first appearance. We left the equation in place to simplify the reading.

Language:

-- "In addition, our approach results quite natural in terms of biological plausibility.": ungrammatical

We changed the text. "results quite" -> "turns out to be very"

-- "combined to a deterministic generation of the spikes": combined --> compared ?

We changed the text in "together with".

-- "bringing the neuron further from the threshold and ensuring a safety gap": also called margin

We changed the text.

Reviewer 3

Reviewer #3: The authors carefully addressed all the comments of the previous review round.

Some minor issues may be considered:

1. It would be nice to mention full-FORCE approach in the introduction in more explicit manner, since that is the most closely related algorithm published so far.

We included the following text in the introduction:

"*The choice of a target-based learning protocol was already described in \cite{Ingrosso2019,depasquale2018full} as the full-FORCE algorithm. The major difference of our model is that we propose as a target a specific spatio-temporal spike pattern, allowing for precise spike timing coding.*"

2. Not all of the source code is provide, e.g. coBa model is missing

We now included the code for the coBa in the github repository.

some typos:

line 205 parenthesis

We corrected the text.

line 312 "The in the case"

We corrected the text.
